# Esrrγa regulates nephron and ciliary development by controlling prostaglandin synthesis

**DOI:** 10.1242/dev.201411

**Published:** 2023-05-26

**Authors:** Hannah M. Wesselman, Ana L. Flores-Mireles, Aidan Bauer, Liming Pei, Rebecca A. Wingert

**Affiliations:** ^1^ Department of Biological Sciences, Center for Stem Cells and Regenerative Medicine, Center for Zebrafish Research, Boler-Parseghian Center for Rare and Neglected Diseases, Warren Center for Drug Discovery, University of Notre Dame, Notre Dame, IN 46556, USA; ^2^ Department of Biological Sciences, Eck Institute for Global Health, University of Notre Dame, Notre Dame, IN 46556, USA; ^3^ Center for Mitochondrial and Epigenomic Medicine, Department of Pathology and Laboratory Medicine, Children's Hospital of Philadelphia, Philadelphia, PA 19104, USA; ^4^ Department of Pathology and Laboratory Medicine, Perelman School of Medicine, University of Pennsylvania, Philadelphia, PA 19104, USA

**Keywords:** Cilia, Kidney, Nephron, Multiciliated cell, Differentiation, *esrrγa*, *ptgs1*, Prostaglandin, Zebrafish

## Abstract

Cilia are essential for the ontogeny and function of many tissues, including the kidney. Here, we report that transcription factor ERRγ ortholog estrogen related receptor gamma a (Esrrγa) is essential for renal cell fate choice and ciliogenesis in zebrafish. *esrrγa* deficiency altered proximodistal nephron patterning, decreased the multiciliated cell populace and disrupted ciliogenesis in the nephron, Kupffer's vesicle and otic vesicle. These phenotypes were consistent with interruptions in prostaglandin signaling, and we found that ciliogenesis was rescued by PGE_2_ or the cyclooxygenase enzyme Ptgs1. Genetic interaction revealed that peroxisome proliferator-activated receptor gamma, coactivator 1 alpha (Ppargc1a), which acts upstream of Ptgs1-mediated prostaglandin synthesis, has a synergistic relationship with Esrrγa in the ciliogenic pathway. These ciliopathic phenotypes were also observed in mice lacking renal epithelial cell (REC) ERRγ, where significantly shorter cilia formed on proximal and distal tubule cells. Decreased cilia length preceded cyst formation in REC-ERRγ knockout mice, suggesting that ciliary changes occur early during pathogenesis. These data position Esrrγa as a novel link between ciliogenesis and nephrogenesis through regulation of prostaglandin signaling and cooperation with Ppargc1a.

## Teaser

**Summary:** Understanding of ciliopathy disorders has been advanced by the discovery that the orphan nuclear receptor Esrrγa regulates kidney organogenesis and ciliary development in zebrafish and the mouse.

## INTRODUCTION

Cilia are hair-like organelles that project from the surface of nearly every quiescent vertebrate cell, where they serve crucial sensory and signal transduction functions. Cilia can be either motile or non-motile, and are comprised of a microtubule-based axoneme that is anchored by a basal body via a connecting transition zone (Fliegauf et al., 2007; Satir and Christensen, 2007). Ciliated cells possess either a single primary cilium, or have bundles with dozens to hundreds of cilia, the latter being aptly named ‘multiciliated cells’ (MCCs) (Choksi et al., 2014; Lewis and Stracker, 2021). During development, cilia are essential for the establishment and maintenance of planar cell polarity and the organization of essential signaling molecules. Aberrant ciliogenesis results in disease states that affect the kidney, liver, pancreas, retina, reproductive and nervous systems (Pazour et al., 2020). Cilia defects have been linked to kidney disorders such as polycystic kidney disease (PKD), Bardet-Biedl syndrome, Joubert syndrome and others (McConnachie et al., 2020). Etiologies for these conditions vary, but many arise due to defects in ciliary proteins. Production of healthy cilia also requires proper control of the ciliogenic transcriptional program. Such regulators include the RFX family of transcription factors, which are essential for primary and motile cilia formation, and interactions with Foxj1 that can further regulate the development of motile cilia (Hellman et al., 2010; Thomas et al., 2010; Wallmeier et al., 2019; Yu et al., 2008). The hepatocyte nuclear factor 1B (HNF1B) also regulates several ciliary genes, thereby contributing to kidney development and/or disease progression (Chambers and Wingert, 2020a; Clissold et al., 2015; Gresh et al., 2004; Hiesberger et al., 2005; Naylor et al., 2013; Sander et al., 2019; Thomas et al., 2010).

Recent studies have continued to identify additional genetic pathways that are essential for ciliogenesis as well as renal function and development. Among these, the transcriptional coactivator encoded by *peroxisome proliferator-activated receptor (PPAR) gamma, coactivator 1 alpha* (*ppargc1a*; known as PGC1α in mammals) regulates kidney function through mitochondrial biogenesis, and ciliogenesis through control of prostanoid production (Chambers et al., 2020b; Chambers and Wingert, 2020b; Fontecha-Barriuso et al., 2020). Ppargc1a promotes the biosynthesis of prostaglandin E_2_ (PGE_2_) by inducing expression of *prostaglandin-endoperoxide synthase 1* (*ptgs1*; also known as *cox1*) in the adult mammalian kidney and zebrafish embryo kidney (Chambers et al., 2020b; Tran et al., 2011). In turn, PGE_2_ is required for proper ciliary outgrowth by modulating intraflagellar transport (IFT) and terminal epithelial differentiation (Jin et al., 2014; Jin and Zhong, 2022; Marra et al., 2019a). Interestingly, *ppargc1a* expression and PGE_2_ production are both required for proper development of kidney functional units, known as nephrons, where they influence patterning of nephron tubule segments to mitigate the fate choice between MCC and transporter cell lineages (Chambers et al., 2018, 2020b; Marra et al., 2019a; Poureetezadi et al., 2016). Further, agonists of PGE_2_ receptors were recently demonstrated to alleviate severe renal dysfunction in the ciliopathy nephronophthisis (NPH) (Garcia et al., 2022).

ERRγ, an orphan nuclear receptor, has been found to interact with both HNF1B and PGC1α in multiple contexts. ERRγ and HNF1B cooperate to regulate mitochondrial function and proximal kidney cell development (Sander et al., 2019; Zhao et al., 2018). Similarly, ERRγ and PGC1α bind common hormone response elements in kidney cells, and work synergistically in mitochondrial biogenesis in various cell types (Fan et al., 2018; Finck and Kelly, 2006; Liu et al., 2005; Wang et al., 2008a). Phenotypes observed in *Esrrγ* knockout (KO) models further support its role in the regulation of energy production, as tissues with high energy demand, including the heart and kidney, are dysregulated. Specifically, *Esrrγ* KO mice die soon after birth, and the renal tissue of these mice has decreased ureteric branching (Alaynick et al., 2007, 2010; Berry et al., 2011). Furthermore, the kidney-specific ERRγ murine KO results in kidney cysts with abnormal nephron function (Zhao et al., 2018), and chromosomal translocation of the *ERRγ* locus in humans is associated with bilateral renal agenesis/hypoplasia/dysplasia (Harewood et al., 2010). Collectively, these findings suggest that ERRγ plays multiple roles in kidney development, yet the roles of this factor in nephrogenesis and ciliary development have not been explored until the present study.

Here, we report that *esrrγa* (also known as *esrrga*) is necessary for nephron segmentation and ciliogenesis. In the zebrafish embryonic kidney, or pronephros, genetic deficiency of *esrrγa* resulted in cell patterning defects and a decreased number of MCCs. Cilia on MCCs and primary epithelial cells were also significantly shortened in renal and non-renal populations of *esrrγa*-deficient animals. These characteristics were strikingly reminiscent of prostaglandin signaling defects during early development. Consistent with this, *esrrγa*-deficient animals had low PGE_2_ and attenuated expression of the cyclooxygenase enzyme encoded by *ptgs1* – and their ciliary defects were rescued by PGE_2_ or Ptgs1. We found that genetic interaction of *esrrγa* and *ppargc1a* promotes *ptgs1* expression to drive PGE_2_ biosynthesis, and thereby ciliogenesis. Finally, using the renal epithelial cell (REC) ERRγ KO mouse line, we discovered that mice lacking ERRγ exhibited shortened cilia across tubule segments, even before cystogenesis. Taken together, these findings provide fundamental new insights about the regulatory networks that direct ciliated cell development and pronephros ontogeny upstream of prostaglandin signaling.

## RESULTS

### *esrrγa* is expressed in renal progenitors

Previous research has demonstrated that *Esrrγ* (*ESRRG*) is expressed in the mouse and human kidney, with particularly high expression profiles in the loop of Henle (RID: N-GK5G, 2-5CE6, 2-5CEA, 16-5WSW) (Harding et al., 2011; Lindström et al., 2021; McMahon et al., 2008). Owing to evolutionary whole genome duplication events, zebrafish have two homologs for *Esrrγ* – *esrrγa* and *esrrγb* (Tohmé et al., 2014). Of these, only *esrrγa* is specifically expressed in a pattern consistent with its localization to renal progenitors and later the distal nephron region, whereas *esrrγb* is not spatially restricted through early developmental stages (Bertrand et al., 2007; Thisse and Thisse, 2008).

**Fig. 1.**
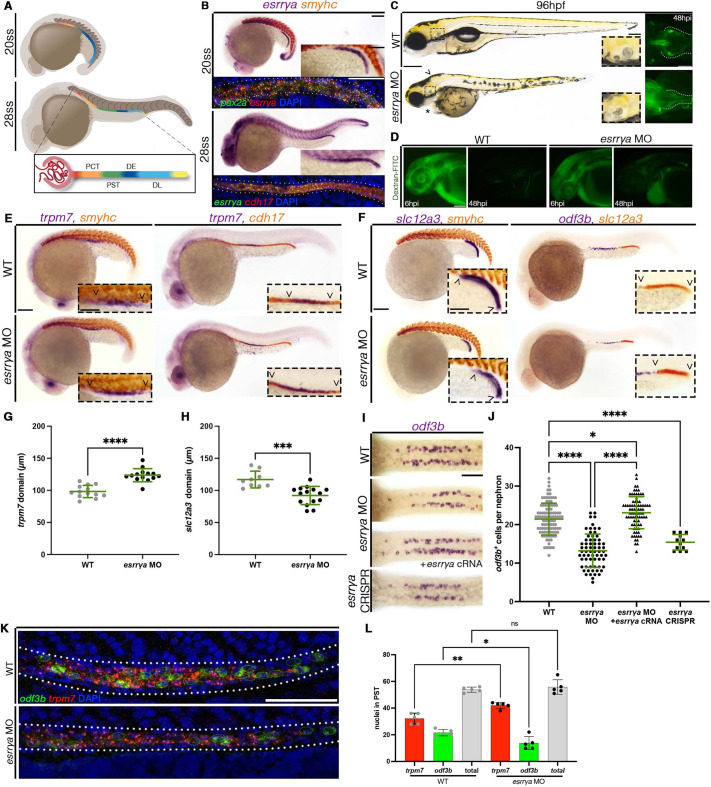
***esrrγa* is essential for nephrogenesis.** (A) Zebrafish nephron development from 20-28 ss. Nephrons possess proximal convoluted tubule (PCT), proximal straight tubule (PST), distal early (DE) and distal late (DL) segments. (B) WISH and FISH reveal that *esrrγa* transcripts colocalize with renal progenitor marker *pax2a* at 20 ss. Somites marked with *smyhc* (red). At 28 ss, *esrrγa* colocalizes with tubule marker *cdh17*. (C) *esrrγa* morphants display pericardial edema (asterisk), smaller eyes and altered head morphology (arrowhead) and fused otoliths (inset) (left). Dextran-FITC in the PCT at 48 hpi, white dotted line outlines nephron (right). (D) WT and *esrrγa* SB morphant at 6 hpi and 48 hpi after dextran-FITC. (E,F) WISH for PST marker *trpm7* (E) and DL marker *slc12a3* (F) with somite marker *smyhc* or nephron marker *cdh17*. (G,H) Length of PST (G) and DL (H) at 28 ss, each dot represents a single animal. (I) WISH for MCC marker *odf3b* at 24 hpf. (J) MCCs per nephron; each dot represents one nephron. Two nephrons were counted per animal. (K) FISH for MCCs (*odf3b*, green and dashed ovals), PST cells (*trpm7*, red) and DNA (DAPI, blue) at 24 hpf. (L) Absolute cell number of *odf3b*- and *trpm7*-expressing cells at 24 hpf. Each dot represents a single animal. Data are mean±s.d. *P<0.05, **P<0.01, ***P<0.001, ****P<0.0001 (unpaired *t*-test or one-way ANOVA). ns, not significant. Scale bars: 100 µm (B-F); 50 µm (B,C,E,F insets, I,K).

To further assess the expression of *esrrγa* during pronephros ontogeny, we performed whole-mount *in situ* hybridization (WISH) in wild-type (WT) zebrafish embryos. As renal progenitors are patterned into distinct segments by the 28 somite stage (ss) (Fig. 1A) (Wingert, et al., 2007; Wingert and Davidson, 2011), we assessed *esrrγa* expression between the 5 and 28 ss. *esrrγa* transcripts were detected in a pattern consistent with their location within the bilateral stripes of renal progenitors at the 8 ss, and the nephron distal tubule segments at the 28 ss (Fig. 1B; Fig. S1A,B). We also detected *esrrγa* transcripts in the region of the Kupffer's vesicle (KV), a transient ciliated organ responsible for left-right patterning, at approximately both the 10 ss and 15 ss (Fig. S1B). Double WISH revealed that *esrrγa*^+^ cells also expressed the essential kidney transcription factor *pax2a* at the 10 ss and nephron marker *cdh17* at the 28 ss (Fig. S1C). This co-expression was further confirmed with fluorescent *in situ* hybridization (FISH), which showed that *esrrγa* transcripts were detected in *pax2a^+^* renal cells through the 20 ss, and *cdh17^+^* cells at the 28 ss (Fig. 1B; Fig. S1D,E). Given this expression pattern throughout renal progenitor development, we hypothesized that *esrrγa* may have roles in nephrogenesis.

### *esrrγa* is required for nephron segmentation

In the developing mouse kidney, ERRγ KO disrupts branching morphogenesis and renal papilla formation, and causes perinatal lethality; in addition, kidney-specific ERRγ KO causes renal cyst formation (Berry et al., 2011; Zhao et al., 2018). To interrogate the function of *esrrγa* during zebrafish pronephros development, we performed loss-of-function studies using a variety of models. First, we validated a previously published splice-blocking morpholino oligonucleotide (MO) (Tohmé et al., 2014). Using reverse transcription polymerase chain reaction (RT-PCR), we confirmed that the MO blocked the exon 1 splice donor site, whereby a portion of exon 1 was excised and produced a transcript with a premature stop codon (Fig. S2A,B,D).

Compared with WT embryos, *esrrγa* morphants displayed several phenotypes, notably pericardial edema and otolith malformations at 96 h post fertilization (hpf) (Fig. 1C), which are suggestive of renal and ciliary defects (Kroeger et al., 2017; Swanhart et al., 2011). Despite these morphological differences, *esrrγa*-deficient animals initially developed features of a normal body plan and body length at 24 hpf (Fig. S2E). *esrrγa* morphants exhibited characteristics of aberrant fluid homeostasis as development progressed based on the appearance of fluid retention around the heart, but did not exhibit accumulations in other locations, such as pronephric cysts. Thus, we hypothesized that *esrrγa* loss of function led to dysregulated fluid and waste clearance. To test this, we performed fluid clearance assays in 48 hpf *esrrγa* morphants and their WT siblings by provision of a fluorescent dextran conjugate and tracked the fluorescence over time. The proximal tubule in WT and morphant nephrons was able to uptake the conjugate, as evidenced by the accumulation of green fluorescence in the proximal segments (Fig. 1C). This indicated that glomerular blood filtration and proximal tubule bulk reabsorption via endocytosis were normal (Anzenberger et al., 2006). Over time, WT animals were able to clear the conjugate from the body, as the fluorescence significantly decreased between the two timepoints throughout the head, trunk and pericardium (Fig. 1D; Fig. S2F). In contrast, however, *esrrγa* morphants exhibited a significantly higher level of fluorescence in these regions at the 48 h post injection (hpi) timepoint, which indicated less fluid clearance over time compared with WT controls (Fig. 1D; Fig. S2F). Taken together, these data indicate that *esrrγa* morphants exhibit decreased nephron fluid clearance over time compared with WT. This led us to hypothesize that nephrogenesis and/or ciliated cell formation were compromised in *esrrγa*-deficient embryos. Therefore, we next assessed *esrrγa* loss of function on each nephron segment and distinct ciliated cell populations within the kidney.

The zebrafish embryonic nephrons are comprised of a blood filter followed by two proximal and two distal tubule segments (Fig. 1A) (Wingert et al., 2007). We found that, upon knockdown of *esrrγa*, the proximal straight tubule segment (PST, marked by *trpm7*) was expanded, whereas the distal late segment (DL, marked by *slc12a3*) was decreased in length at both the 20 ss and 28 ss (Fig. 1E,F). The proximal convoluted tubule segment (PCT, marked by *slc20a1a*), the distal early segment (DE, marked by *slc12a1*) and the overall length of the nephron tubule (marked by *cdh17*) remained unchanged (Fig. S3A,C-E). The PCT also exhibited successful proximal migration towards the glomerulus and the correct convoluted morphology by 3 days post fertilization (dpf) (Fig. S3G). The observed composition changes were notable as early as the 20 ss and were not a result of changes in cell proliferation, cell death or total cell number (Fig. 1G,H; Fig. S3H-O). This supports the notion that Esrrγa operates early and specifically throughout nephron formation, as the pattern of distinct segments are altered.

**Fig. 2.**
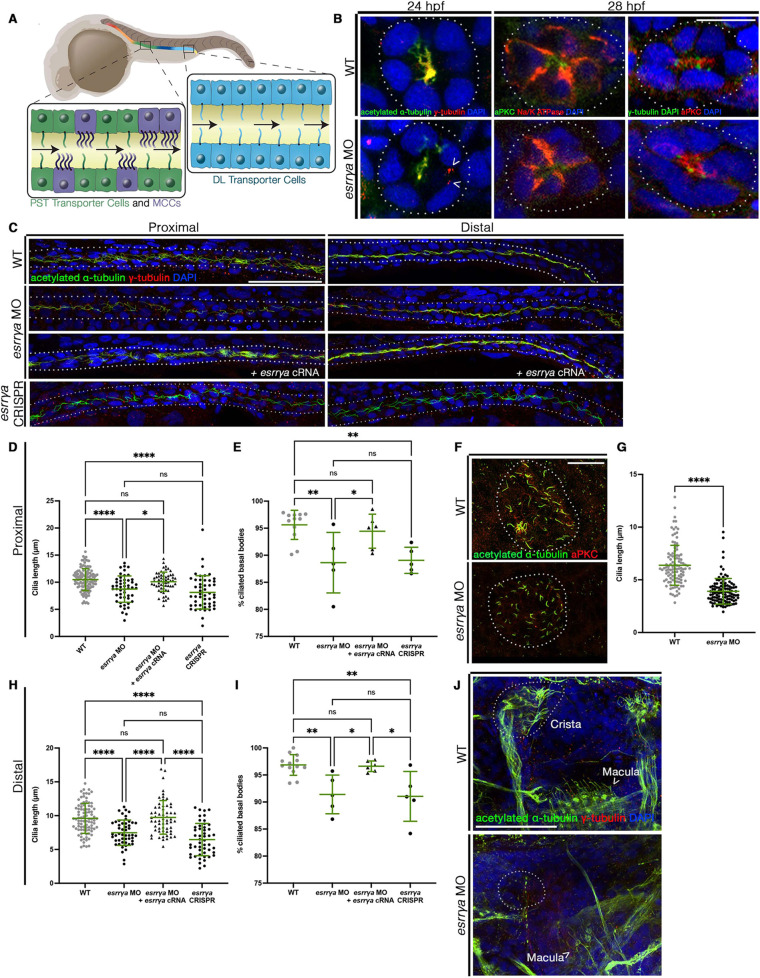
***esrrγa* is essential for ciliogenesis.** (A) Multiciliated cells (MCCs) and monociliated transporter cells in the proximal straight tubule (PST) and distal late (DL). (B,C) IF for indicated markers. Dotted lines indicate the nephron. (D,H) Cilia length. Each dot represents one cilium, and ten cilia were measured per animal. WT *n*=12, *esrrγa* MO *n*=5, *esrrγa* MO with cRNA *n*=6, *esrrγa* crispant *n*=5. (E,I) Percentage of ciliated basal bodies. Each dot represents one animal. WT *n*=12, *esrrγa* MO *n*=5, *esrrγa* MO with cRNA *n*=6, *esrrγa* crispant *n*=5. (F) IF for indicated markers in Kupffer's vesicle (KV; outlined with dotted line) at the 10 ss. (G) KV cilia length. Each dot represents a single cilium. WT *n*=7, *esrrγa* MO *n*=7. (J) IF for indicated markers in ear at 4 dpf. Arrowhead denotes macula cilia, dotted line surrounds cristae structures. Data are mean±s.d.. *P<0.05, **P<0.01, ****P<0.0001 (unpaired *t*-test or one-way ANOVA). ns, not significant. Scale bars: 10 µm (B); 25 µm (F); 50 µm (C,J).

To further explore the mechanics of these segment changes, we studied another cell type present within the nephron, MCCs, which are intermingled with transporter cells in the PST segment (Fig. 2A). Preceding work has found that increased monociliated transporter cell identity can be associated with a coordinated decrease in MCC identity (Chambers et al., 2020b; Marra et al., 2019a,b). Indeed, we found that *esrrγa* deficiency resulted in a decreased MCC cell number [marked by *odf3b* (also known as *cimap1b*), an established marker of terminally differentiated MCCs], and co-injection of *esrrγa* RNA was sufficient to rescue the splice-blocking morpholino (Fig. 1I,J) (Barrodia et al., 2018; Li et al., 2014; Liu et al., 2007; Xie et al., 2020). FISH analysis of the PST domain (marked by the boundaries of *trpm7*) also revealed a shift towards a transporter cell identity (Fig. 1K). Although the overall average cell number (calculated using DAPI staining) in this domain did not change, there was an increase in the number of *trpm7*^+^ cells accompanied by a coordinated decrease of *odf3b*^+^ cells (Fig. 1L). MCC precursors were also affected in *esrrγa*-deficient animals, based on a significant decrease in the number of *jag2b*-expressing cells (Fig. S3B,F).

Next, we assessed the effects of *esrrγa* deficiency with several independent models, first assessing an *esrrγa* MO that blocks protein translation (*esrrγa* ATG MO) (Tohmé et al., 2014) (Fig. S2A). *esrrγa* ATG morphants exhibited similar phenotypes to those injected with the *esrrγa* splice-blocking MO, including malformed otoliths and alterations in fluid homeostasis, as evidenced by fluid accumulation around the heart (Fig. S4A). We also observed a decrease in the number of MCCs in ATG morphants (Fig. S4B,C). Furthermore, we tested *esrrγa* loss of function using CRISPR-Cas9 genome editing, whereby WT embryos were microinjected with a cocktail of two guide RNAs that targeted exon 1 of *esrrγa* (Fig. S2A) (Chambers et al., 2020a). The crispants were validated by a T7 endonuclease assay (Fig. S2C). *esrrγa* crispants also recapitulated the observed morpholino phenotypes, including alterations to fluid homeostasis by 96 hpf and a decrease in MCC number (Fig. 1I,J; Fig. S2I). In sum, these findings led us to conclude that *esrrγa* is essential to mitigate cell fate decisions between the MCC and monociliated transporter cell identity.

### *esrrγa* is required for ciliogenesis in the kidney and other tissues

Ciliogenesis is a complex process, requiring proper basal body production around the centrioles, amplification of the basal bodies (in the case of MCCs), basal body docking at the apical surface, and finally cilia outgrowth, mediated by anterograde and retrograde intraflagellar transport (Spassky and Meunier, 2017). Previous studies in the zebrafish pronephros have found that decreased MCC number can be associated with aberrations in ciliogenesis (e.g. decreased cilia outgrowth) (Chambers et al., 2020b; Marra et al., 2019a; Wesselman et al., 2023a).

Considering the observed decrease in MCC number in *esrrγa*-deficient animals, we investigated whether cilia formation was affected. As ciliopathic phenotypes were observed across all of the *esrrγa*-deficient animals, we first evaluated the *esrrγa* splice-blocking morpholino model, as its penetrance was upwards of 90%. Several pronephros segments are comprised of MCCs interspersed amongst monociliated transporter cells (Fig. 2A). We performed immunofluorescence (IF) to assess epithelial cell polarity and ciliogenesis. *esrrγa*-deficient animals established normal polarity at 28 hpf, as both apical (aPKC) and basolateral (Na^+^K^+^ ATPase) proteins were correctly localized (Fig. 2B) (Gerlach and Wingert, 2014). In addition, we observed that basal bodies (γ-tubulin) were docked at the apical surface, as they colocalized with the apical membrane (aPKC) (Fig. 2B). IF of 24 hpf morphants analyzed for cilia structures [cilia (α-tubulin), basal bodies (γ-tubulin)] revealed that, even though basal bodies were correctly localized, cilia were decreased (Fig. 2B). Furthermore, some basal bodies observed in *esrrγa*-deficient animals were not associated with a cilium projection (Fig. 2B). These data suggested that *esrrγa* contributes to ciliogenesis independently of polarity establishment or basal body docking.

To further explore the role of *esrrγa* in cilia outgrowth, we used IF to mark cilia (α-tubulin), basal bodies (γ-tubulin) and DAPI in whole mounts of *esrrγa*-deficient animals and WT siblings. This was followed by confocal imaging of both the proximal and distal pronephros to capture cilia protruding from MCCs as well as transporter cells, respectively. Using established protocols to quantify cilia structure (Chambers et al., 2020b; Wesselman et al., 2023b), we found that cilia were disrupted in both the proximal and distal pronephros of splice-blocking morphants (Fig. 2C). In particular, ciliary length was significantly shorter in *esrrγa*-deficient animals compared with WT (Fig. 2D,H). We did not observe significant changes in the number of basal bodies or in cell number (Fig. S6A-D). However, *esrrγa* morphants had fewer ciliated basal bodies compared with WT controls (Fig. 2E,I). To further test the specificity of our splice-interfering morpholino, we co-injected animals with *esrrγa* capped RNA (cRNA). Similar to MCC number, supplementation of mature *esrrγa* transcript alongside the morpholino was sufficient to rescue cilia length and ciliated basal bodies (Fig. 2C-E,H,I). Furthermore, these ciliary defects were recapitulated in both the *esrrγa* ATG morphants (Fig. S4D-N) and the *esrrγa* crispants (Fig. 2C-E,H,I). From these data, we concluded that *esrrγa* deficiency interferes with cilia formation in both MCCs and RECs.

In addition to the kidney, cilia are crucial to several other tissues across vertebrates. In the zebrafish this includes, but is not limited to, the KV (the early left-right organizer) and the otic vesicle (ear structure). To determine whether *esrrγa* operates solely in the pronephros, we next investigated the effect of *esrrγa* deficiency on these other tissues. Given the consistency of the ciliopathic phenotypes (e.g. decreased cilia length) across all of the deficiency models, we chose to evaluate cilia formation in other tissues using the *esrrγa* splice-blocking morpholino, as this method is the highest throughput. The observed expression of *esrrγa* in the KV made this transient organ of particular interest, so we used IF to mark the KV using aPKC (apical surface) and α-tubulin (cilia) in both *esrrγa* morphants and WT siblings at the 10 ss (Fig. 2F). Like the pronephros, cilia length was significantly reduced in the KV of *esrrγa*-deficient animals (Fig. 2G). As the KV is responsible for embryonic patterning, we evaluated whether the decreased cilia length resulted in other morphological defects. In particular, we found that heart looping in *esrrγa* morphants was significantly altered, as ∼20% of animals displayed either *situs inversus* or *mid* phenotypes of the heart marker *myl7* (Fig. S2G,H). We also used IF to identify cilia and basal bodies in the ear at 4 dpf. *esrrγa* morphants exhibited decreased fluorescence of α-tubulin in the region of both macula and cristae structures, the latter of which was nearly absent altogether (Fig. 2J). These data are consistent with ciliary phenotypes observed in the pronephros and suggest that *esrrγa* affects multiple tissues throughout early development.

### *esrrγa* promote ciliogenesis and MCC cell fate by regulating prostanoid biosynthesis

**Fig. 3.**
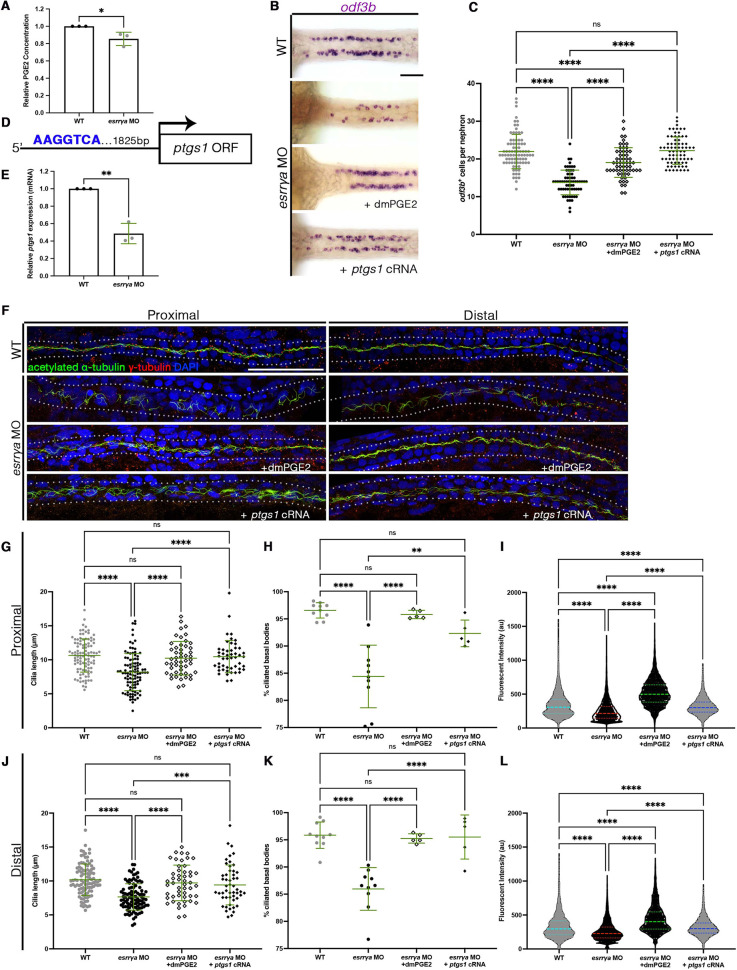
***esrrγa* controls ciliogenesis through regulation of prostaglandin signaling.** (A) Relative PGE_2_ concentration. (B) WISH for MCCs (*odf3b*) at 28 ss. (C) MCCs per nephron. Each dot represents one nephron. Two nephrons were counted per animal. (D) Schematic of a putative Esrrγa binding site upstream of the *ptgs1* open reading frame (ORF). (E) *ptgs1* quantified via qRT-PCR. (F) IF for indicated markers at 28 hpf. Dotted lines indicate nephron tubule. (G,J) Cilia length in proximal (G) and distal (J) pronephros. Each dot represents one cilium. Ten cilia were measured per animal. (H,K) Percentage of ciliated basal bodies in proximal (H) and distal (K) pronephros. Each dot represents a single animal. (I,L) Fluorescent intensity plots (cilia, α-tubulin) for the same relative distance in proximal (I) and distal (L) pronephros. Each dot represents the fluorescent intensity of an animal at a given point across the segment of interest. (G-L) WT *n*=10, *esrrγa* MO *n*=10, *esrrγa* MO with dmPGE_2_ *n*=5, *esrrγa* MO with *ptgs1* cRNA *n*=5. Data are mean±s.d. *P<0.05, **P<0.01, ***P<0.001, ****P<0.0001 (unpaired *t*-test or one-way ANOVA). ns, not significant. Scale bars: 50 µm.

Prostaglandins are formed by the metabolism of arachidonic acid by cyclooxygenase enzymes to form PGH_2_, which can then be further metabolized by prostaglandin synthase enzymes to form prostanoids (Funk, 2001). Early zebrafish embryos contain four prostaglandin signaling molecules (PGE_2_, PGF2α, PGI_2_ and TXA2) (Cha et al., 2005), and essential roles for PGE_2_ have been elucidated in the development and regeneration of kidney, blood and endoderm progenitors (Goessling et al., 2009; Liu et al., 2023; Nissim et al., 2014; North et al., 2007; Poureetezadi et al., 2016). Further, prostaglandin signaling via PGE_2_ is required broadly for vertebrate ciliogenesis, and specifically for renal MCC cell fate choice during zebrafish embryo pronephros development while simultaneously not being sufficient to increase MCC number nor alter cilia formation in WT animals (Chambers et al., 2020b; Jin et al., 2014; Marra et al., 2019a). Interestingly, we noted that *esrrγa*-deficient zebrafish embryos exhibited strikingly similar ciliated cell phenotypes as those with defective PGE_2_ synthesis – namely, decreased MCCs and aberrant cilia (Chambers et al., 2020b; Jin et al., 2014; Marra et al., 2019a; Poureetezadi et al., 2016). Therefore, we hypothesized that perhaps *esrrγa* function was related to a lack of PGE_2_ synthesis. To test this, we used an established ELISA assay to measure endogenous PGE_2_ in WT controls and *esrrγa*-deficient embryos (Esain et al., 2015; Chambers et al., 2020b). Compared with WT, *esrrγa* knockdown resulted in a significant decrease of PGE_2_ (Fig. 3A). This led us to hypothesize that this diminished PGE_2_ level was the basis for the ciliary and cell fate alterations in *esrrγa*-deficient embryos.

To investigate this idea, we examined the consequence of elevating prostanoid levels in in *esrrγa*-deficient embryos. The PGE_2_ analog 16,16-dimethyl-PGE_2_ (dmPGE_2_) has been used widely to study the effects of PGE_2_ because it is a long-acting, stable derivative – unlike PGE_2_ which has a very short half-life (Chambers et al., 2020b; Goessling et al., 2009; Jin et al., 2014; Marra et al., 2019a; Nissim et al., 2014; North et al., 2007; Poureetezadi et al., 2016). We treated WT and *esrrγa*-deficient embryos with dmPGE_2_ and used WISH of *odf3b* to assess MCC cell fate (Fig. 3B). *esrrγa*-deficient embryos treated with 100 µM dmPGE_2_ from shield stage until fixation at 24 hpf had an increase in MCC number compared with their dimethyl sulfoxide (DMSO)-treated siblings, restoring the number similar to that of WT animals (Fig. 3C). As reported in previous studies, dmPGE_2_ was not sufficient to increase MCC number in WT animals (Chambers et al., 2020b; Marra et al., 2019a). Next, we investigated whether dmPGE_2_ was able to restore proper cilia formation. We treated both WT and *esrrγa* morphant animals with 100 µM dmPGE_2_ or vehicle control from shield stage until fixation at 28 hpf and assessed cilia structures using whole-mount IF (Fig. 3F). In both the proximal and distal tubule, dmPGE_2_ rescued cilia length (Fig. 3G,J), ciliated basal bodies (Fig. 3H,K) and corresponding cilia fluorescent intensity (Fig. 3I,L) to WT levels. Together, these data suggest that *esrrγa* interacts with the PGE_2_ pathway to facilitate ciliogenesis and MCC cell fate choice.

Previous research has shown that cyclooxygenase enzymes (Cox1 or Cox2, encoded by *ptgs1*, *ptgs2a/b* in zebrafish, respectively) are crucial for proper ciliogenesis and adoption of the MCC cell identity via the biosynthesis of PGE_2_ (Chambers et al., 2020b; Marra et al., 2019a). Like dmPGE_2_ treatment, overexpression of these enzymes independently is not sufficient to drive ectopic MCC genesis nor alter cilia formation (Chambers et al., 2020b; Marra et al., 2019a). With this in mind, we hypothesized that *esrrγa* may be contributing to cilia formation through *ptgs1*. We first examined the 2 kb promoter region of *ptgs1* for potential binding sites for *esrrγa.* We found one ERR consensus binding motif (AAGGTCA) ∼1.8 kb upstream of the *ptgs1* open reading frame (ORF) (Fig. 3D). It is also worth noting that, unlike estrogen receptors, ERRs can bind DNA and affect transcription as monomers; thus, one consensus sequence can be sufficient to affect expression (Huppunen and Aarnisalo, 2004). To confirm that *esrrγa* deficiency does affect *ptgs1* transcription, we conducted WISH analysis and real-time quantitative reverse transcription PCR (qRT-PCR) of *ptgs1* in *esrrγa*-deficient animals at 24 hpf. The length of the *ptgs1* domain within the pronephros was significantly decreased (Fig. S5A,B), along with the expression level based on qRT-PCR (Fig. 3E). The observed decreased expression of *ptgs1* further supported our hypothesis that *esrrγa* may be contributing to cilia formation through regulation of *ptgs1* transcription*.* Therefore, we sought to determine whether *ptgs1* overexpression alone was sufficient to rescue the effects of *esrrγa* deficiency.

We first examined the effect of co-injection of *ptgs1* RNA with *esrrγa* MO on MCC number (Fig. 3B). Like dmPGE_2_ treatment, *ptgs1* RNA restored MCC number to WT levels (Fig. 3C). We then observed the effect of *ptgs1* overexpression on cilia in both MCCs and mono-ciliated cells using IF (Fig. 3F). In both proximal and distal segments, we found that *ptgs1* cRNA was sufficient to rescue ciliary length (Fig. 3G,J), the number of ciliated basal bodies (Fig. 3H,K), and the corresponding fluorescent intensity of α-tubulin (Fig. 3I,L). Again, these phenotypes were not associated with changes either in net basal body or cell number (Fig. S6A-D). Finally, we also explored whether *esrrγa* overexpression resulted in an elevation of *ptgs1* expression. There was no statistically significant difference in *ptgs1* transcript quantity between WT embryos and those microinjected with *esrrγa* RNA (Fig. S6E). This result is consistent with our previous observation that *esrrγa* overexpression does not elevate MCC number in WT embryos. From these data, we concluded that *esrrγa* promotes PGE_2_ synthesis via *ptgs1* to promote MCC specification and cilia outgrowth in MCC and transporter cell populations.

### *esrrγa* cooperates with *ppargc1a* to control MCC specification and cilia formation

**Fig. 4.**
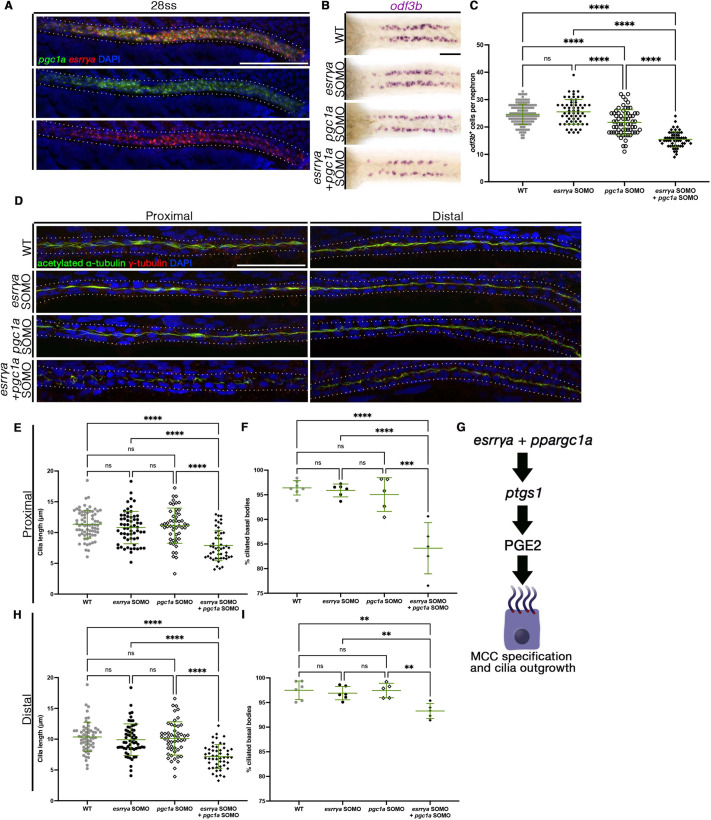
**Genetic interaction of *esrrγa* and *pgc1a* drives MCC specification and ciliogenesis.** (A) FISH reveals that *esrrγa* colocalizes with *pgc1a* in 24 hpf WT zebrafish. (B) MCCs stained via WISH (*odf3b*). (C) MCCs per nephron. Each dot represents one nephron. Two nephrons were measured per animal. (D) IF for indicated markers at 28 hpf. Dotted lines indicate nephron tubule. (E,H) Cilia length in proximal (E) and distal (H) pronephros. Each dot represents one cilium. Ten cilia were measured per animal. WT *n*=6, *esrrγa* SOMO *n*=6, *pgc1a* SOMO *n*=5, *esrrγa* and *pgc1a* SOMO *n*=5. (F,I) Percentage of ciliated basal bodies in proximal (F) and distal (I) pronephros. Each dot represents an individual. (G) *esrrγa and pgc1a* cooperate upstream of *ptgs1* and PGE_2_ production to regulate MCC cell fate and ciliogenesis. Data are mean±s.d. **P<0.01, ****P<0.0001 (one-way ANOVA). ns, not significant. Scale bars: 50 µm.

Recent studies have found that *ppargc1a* is essential for prostaglandin signaling, nephron formation and ciliogenesis (Chambers et al., 2018, 2020b). Interestingly, deficiency of this factor phenocopies the nephron cell fate and MCC defects that we observed in the case of *esrrγa* deficiency (Chambers et al., 2018, 2020b). Specifically, *ppargc1a* deficiency results in decreased MCC number and cilia length, which is caused by decreased expression of *ptgs1* and can be similarly rescued by overexpression of *ptgs1* or dmPGE_2_ treatment (Chambers et al., 2020b). Given these parallels, we tested whether *esrrγa* and *ppargc1a* were expressed in the same cell populations. FISH analysis revealed that *esrrγa* and *ppargc1a* were colocalized within the same pan-distal region of the nephron (Fig. 4A).

Next, we designed genetic interaction studies to explore the relationship between *esrrγa* and *ppargc1a*. Suboptimal morpholinos (SOMOs) have been a consistent and well-established strategy to test whether multiple genes operate synergistically in a pathway in multiple species, including zebrafish and *Xenopus laevis* (Chambers et al., 2020b; Choi et al., 2015; DiBella et al., 2009; Epting et al., 2022; Fowler et al., 2021; Janssens et al., 2013; Kallakuri et al., 2015; Lefkopoulos et al., 2020; Lu et al., 2021; Maerker et al., 2021; Tingler et al., 2022; Wagle et al., 2011). Therefore, we injected SOMO doses of both *esrrγa* and *ppargc1a*, based on previously published doses (Chambers et al., 2018, 2020b). WISH was used to determine changes in MCC number (Fig. 4B; Fig. S7A). *esrrγa* and *ppargc1a* SOMO alone or co-injected with a standard control morpholino resulted in no change, or a slight yet significant decrease, in MCC number when compared with WT, respectively (Fig. 4C; Fig. S7B). However, the combination of both *esrrγa* and *ppargc1a* SOMO together resulted in a significant decrease in MCC number (Fig. 4C). We then interrogated the synergistic effect of *esrrγa* and *ppargc1a* on cilia formation. Similar to MCC number, *esrrγa* and *ppargc1a* SOMO did not appear to have a significant effect on the appearance of cilia, whereas the combination injection showed aberrant cilia structures (Fig. 4D; Fig. S7C). Further, *esrrγa* and *ppargc1a* SOMO injections independently did not significantly change ciliated basal bodies nor cilia length in MCC and transporter cell populations. However, combination of the *esrrγa* and *ppargc1a* SOMO significantly decreased the percentage of ciliated basal bodies as well as cilia length in both pronephric regions of interest (Fig. 4E,F,H,I; Fig. S7F,G,J,K). These changes were not concordant with alterations in basal body or cell number (Fig. S6A-D, Fig. S7D-E,H-I). Overall, this evidence is indicative of a cooperative effect between *esrrγa* and *ppargc1a* in the context of ciliogenesis and MCC specification.

We next sought to determine whether the combined deficiency of *esrrγa* and *ppargc1a* was sufficient to eradicate cilia formation altogether. Upon injection with a full dose of both *esrrγa* and *ppargc1a* morpholino, animals still had MCCs present in the nephron, as well as expression of distal tubule markers (Fig. S8A). Although both the DL length and MCC numbers were significantly decreased (Fig. S8B,C), these levels were similar to that of a single knockdown of either factor independently. Similarly, cilia length and ciliated basal bodies were significantly decreased without affecting the number of cells or basal bodies (Fig. S8D-L). Interestingly, this combined knockdown was not sufficient to eliminate cilia formation in the nephrons altogether. This suggests that, although *esrrγa* and *ppargc1a* cooperate to regulate prostaglandin biosynthesis, there are one or more other independent pathways from prostaglandin signaling that induce MCC fate choice and ciliogenesis (Fig. 4G).

### The role of ERRγ is conserved in murine renal ciliogenesis

**Fig. 5.**
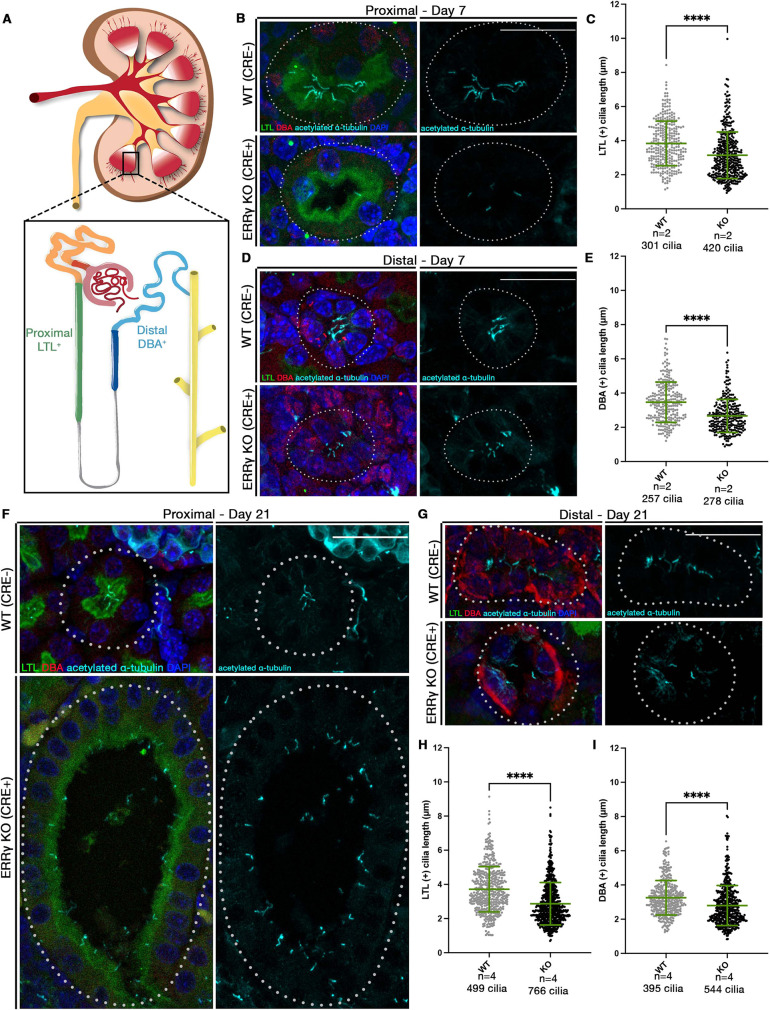
**Murine ERRγ functions in ciliary development.** (A) Schematic of mammalian kidney and nephron. Proximal segments are LTL^+^ and distal segments are DBA^+^. (B,D) IF for α-tubulin in LTL^+^ (B) and DBA^+^ (D) tubules (dotted outline) in 7-day-old animals. (C,E) Cilia length in LTL^+^ (C) and DBA^+^ (E) 7-day old animals. Number of animals given as *n* and number of cilia measured. (F,G) IF for α-tubulin in LTL^+^ (F) and DBA^+^ (G) tubule (dotted outlines) in 21-day-old animals. (H,I) Cilia length in LTL^+^ (H) and DBA^+^ (I) 21-day old animals. Number of animals given as *n* and the number of cilia measured. Data are mean±s.d. ****P<0.0001 (unpaired *t*-test). Scale bars: 25 µm.

Although the zebrafish is an established and conserved model to study renal diseases and ciliopathies, questions remain regarding the conservation of ERRγ function specifically (Corkins et al., 2021; Molinari and Sayer, 2020; Morales and Wingert, 2017). Our current work supports previous research suggesting that ERRγ contributes to kidney function (Zhao et al., 2018). However, ciliogenesis, and specifically renal cilia, were not evaluated in previously generated ERRγ mouse KO lines. To determine whether the function of ERRγ in cilia formation is conserved in the murine kidney, we examined samples from a REC ERRγ KO model (Zhao et al., 2018). These animals appear to be phenotypically normal at birth, but exhibit renal cysts by 3 weeks of age. The mammalian kidney is comprised of nephrons with similar segmentation patterns, where the proximal regions can be marked by staining with Lotus Tetragonolobus Lectin (LTL) and the distal with Dolichos biflorus agglutinin (DBA) (Fig. 5A). Using previously described methods, we used LTL-FITC and DBA-rhodamine to mark the proximal and distal segments, respectively, and cilia were marked with acetylated α-tubulin (Dionne et al., 2018; Jun et al., 2022; Komarynets et al., 2019; Shao et al., 2020; Silva et al., 2019; Verghese et al., 2008, 2009). We first investigated cilia in kidney tubules in 7-day-old animals, when neither ERRγ REC KO animals nor their littermates exhibited cystic phenotypes. Interestingly, we found that cilia were shorter in ERRγ REC KO animals in both proximal (LTL^+^) and distal (DBA^+^) segments (Fig. 5B-E). We next evaluated cilia length in 3-week-old animals, when ERRγ REC KO animals have cysts throughout their kidney (Zhao et al., 2018). Again, we observed shortened cilia in ERRγ REC KO animals in both proximal and distal segments (Fig. 5F-I). As ciliary phenotypes were observed before cystogenesis, when kidneys still appear to be phenotypically similar to their WT siblings, decreased cilia length may be the first sign of pathogenesis in these animals. From these studies, we conclude that ERRγ function appears to be conserved across species, as both mice and zebrafish deficient in ERRγ exhibit reduced ciliary lengths.

## DISCUSSION

Estrogen-related receptors, and specifically ERRγ, have been previously linked to disease states of tissues with high energy demand. Global ERRγ KO mice and cardiac-specific overexpression mice exhibit early lethality (Alaynick et al., 2007, 2010; Lasheras et al., 2021). Similar dysfunction is seen in the kidney, as ERRγ KO results in deficient ureteric branching, kidney cysts, and decreased mitochondrial function and solute transportation (Berry et al., 2011; Zhao et al., 2018). In humans, mutations in or decreased expression of *ERRγ* have been linked to incidence of congenital anomalies of the kidney and urinary tract (CAKUT) and chronic kidney disease (CKD) (Berry et al., 2011; Eichner and Giguère, 2011; Harewood et al., 2010; Hock and Kralli, 2009; Misra et al., 2017; Zhao et al., 2018). Until the current work, however, the mechanism by which ERRγ contributes to ciliated cell development had not been explored.

Our findings suggest that *esrrγa* works with *ppargc1a* upstream of prostaglandin signaling to facilitate nephron cell development and ciliogenesis. Specifically, *esrrγa* is required to adopt the MCC fate, and its absence leads to decreased MCC number with a coordinated increase of PST transporter cells. In addition, *esrrγa* knockdown resulted in decreased ciliated basal bodies and decreased cilia length in both mono- and multiciliated cell populations in the nephron and other tissues. The observed decreased MCC number and aberrant cilia could be rescued by co-injection of *ptgs1* or treatment with dmPGE_2_, showing for the first time that *esrrγa* works upstream of prostanoid production. Furthermore, genetic interaction studies of *esrrγa* and *ppargc1a* resulted in phenotypes reminiscent of knockdown of each gene independently, with decreased MCC number and atypical cilia. Aberrant renal cilia formation was also observed in ERRγ-deficient mice, suggesting conservation of ERRγ function across vertebrates. Together, these data deepen our understanding of ciliated cell development.

Prostaglandins have been established as key bioactive molecules in various tissues, and implicated in disease states relating to inflammation, vascular development, cardiac injury, regeneration and kidney disease (FitzSimons et al., 2020; Lannoy et al., 2020; Liu et al., 2023; Marra et al., 2019a; Sparks and Coffman, 2010; Ugwuagbo et al., 2019). In some models, blockade of a prostaglandin receptor (EP4) can improve cystic disease states, yet PGE_2_ has also been implicated as an essential factor of cilia outgrowth and proper nephron patterning, which together point to the importance of proper spatiotemporal control of prostaglandin dosage (Chambers et al., 2020b; Jin et al., 2014; Lannoy et al., 2020; Marra et al., 2019a; Poureetezadi et al., 2016). Most recently, PGE_2_ receptor agonists were found to mitigate defective ciliogenesis in several models of the ciliopathy NPH, positioning these and similar agents as a possible therapeutic option for juvenile NPH-associated ciliopathies (Garcia et al., 2022). Here, we have added to that growing body of knowledge, as dmPGE_2_ and *ptgs1* were able to rescue both cell-type (MCC deficiency) and ciliopathic (ciliated basal bodies, and cilia length) phenotypes in *esrrγa* deficiency. The dmPGE_2_ treatment was unable to rescue the DL length (Fig. S9). However, a restoration of the DL was not anticipated as exogenous dmPGE_2_ and prostaglandin inhibition have been shown to result in the same DL domain decrease (Poureetezadi et al., 2016). These somewhat contradictory findings speak to the importance of precise PGE_2_ dosage, and also suggest that *esrrγa* may control segmentation through a mechanism independent of prostaglandin signaling. Further research is required to interrogate the mechanism by which the distal tubule segment is regulated. Candidate transcription factors like *tbx2b* may be of interest, as it operates downstream of *ppargc1a* in establishing distal cell fate identity (Chambers et al., 2018; Drummond et al., 2017), as well as *mecom*, *tbx2a*, *emx1*, *gldc* and *esr2b*, which are also essential for distal segment fate (Drummond et al., 2017; Li et al., 2014; Morales et al., 2018; Weaver et al., 2022; Wesselman et al., 2023c). Although continued kidney research in animal models such as zebrafish affords the opportunity to dissect gene functions and model renal diseases like ciliopathies, parallel studies in mammalian models are also essential (Corkins et al., 2021; McKee and Wingert, 2015; Molinari and Sayer, 2020; Morales and Wingert, 2017).

*esrrγa* and *ppargc1a* act similarly upstream of nephron and ciliogenesis in zebrafish, and we found a clear synergistic relationship between these factors within developing renal progenitors. Whether this synergism exists in other tissues remains important to resolve. Interestingly, ciliogenesis is not the first context in which these factors have been linked, and some have even suggested that PGC1a acts as a protein ligand of ERRγ (Audet-Walsh and Giguére, 2015). Both *esrrγa* and *ppargc1a* have been independently implicated in mitochondrial function and various disorders, including diabetes and kidney disease (Audet-Walsh and Giguére, 2015; Chambers and Wingert, 2020a,b; Guo et al., 2015; Ishimoto et al., 2017; Knutti and Kralli, 2001; Long et al., 2016; Misra et al., 2017; Poidatz et al., 2012; Sharma et al., 2013; Zhao et al., 2018). Our present studies have shown a strong and consistent interaction between *esrrγa* and *ppargc1a*, but cilia structure and specific nephron cell types were not evaluated before now. Although we have begun filling this gap in knowledge through our SOMO combination experiments, future work is needed to elucidate the molecular nature of this relationship. Furthermore, the relationship between mitochondrial function and ciliogenesis remains an important and unresolved topic for subsequent investigation. Interestingly, mitochondrial function regulated via *ppargc1a* has been recently linked to ciliogenesis in motile monociliated cells in the zebrafish (Burkhalter et al., 2019). Additional studies suggest that ciliary signaling channels may, in turn, regulate mitochondrial function in KV cells and proximal kidney cells (Fujii et al., 2021; Hofherr et al., 2018). With the current body of knowledge in mind, future studies are needed to interrogate whether Errγa and Ppargc1a also co-regulate mitochondrial biogenesis as well as the relationship between the ciliogenic program of MCCs and mitochondria health.

In terms of the regulation of cilia outgrowth, it is not yet known whether Errγa and Ppargc1a directly bind to one another in the promoter or enhancer region of *ptgs1* or regulate ciliogenesis through some other mechanism. As both Errγ and Ppargc1a have been shown to interact with Hnf1b in the context of kidney tissue, especially in ciliopathic conditions, it is possible that Hnf1b may act as the link in this synergistic relationship (Casemayou et al., 2017; Verhave et al., 2016; Zhao et al., 2018). Furthermore, neither *esrrγa* or *ppargc1a* deficiency alone is sufficient to eradicate all pronephric MCCs and cilia. This may be due to redundant function of *esrrγb*, as *esrrγa/b* function redundantly in the development of the otic vesicle (Tohmé et al., 2014). Alternatively, maternal deposition of either of these factors could explain the basal level of cilia production, or perhaps the presence of other ciliogenic factors is sufficient to compensate for *esrrγa* and *ppargc1a* loss to drive low levels of ciliogenesis*.* Future studies may be interested in the interaction between *esrrγa/ppargc1a* and other known factors that regulate MCC development and/or ciliogenesis (e.g. *e2f4/5*, *etv5a*, *foxj1a*, *gmnc*, *irx2a, mcidas*, *mecom*, *multicilin*, *myb*, Notch, *rfx2*) (Chong et al., 2018; Dubruille et al., 2002; Li et al., 2014; Liu et al., 2007; Ma and Jiang, 2007; Marra et al., 2016, 2019b; Marra and Wingert, 2016; Stubbs et al., 2012; Tan et al., 2013; Xie et al., 2020; Yu et al., 2008; Zhou et al., 2015, 2020). Our data at present do not position *esrrγa* or *ppargc1a* as ‘master regulators’ of ciliogenesis or MCC fate choice, given that deficiency of these factors leads to reductions as opposed to abrogation of cilia or MCCs, respectively.

The link between ciliopathies and aberrant kidney structure and function has long been established (Wang and Dong, 2013; Winyard and Jenkins, 2011). However, cilia of kidney epithelial cells can present a dynamic and diverse range of phenotypes during disease progression (Kim et al., 2013; Miyoshi et al., 2011; Park, 2018; Verghese et al., 2008, 2009; Wang et al., 2008b; Wann and Knight, 2012). In some cystic conditions, like PKD and NPH, cilia appear to lengthen (Garcia et al., 2022; Shao et al., 2020). Conversely, shortened cilia are observed with centrosome disruption, accumulation of ROS or, soon after, ischemic injury (Dionne et al., 2018; Han et al., 2016; Kim et al., 2013; Verghese et al., 2008). Detection of shed ciliary components, like acetylated α-tubulin, in urine has also been recently proposed as marker for kidney disease (Han et al., 2016; Kim et al., 2013; Park, 2018). Here, we provide new insights regarding the nexus of nephron and ciliary development, as *esrrγa* regulates prostaglandin signaling through cooperation with *ppargc1a*. Considering that both mice and zebrafish with deficient ERRγ exhibit aberrant renal cilia, our study has broad implications for ciliogenesis across species and warrants further investigation into shortened cilia as a sign of pathogenesis.

## MATERIALS AND METHODS

### Experimental models and subject details

The Center for Zebrafish Research at the University of Notre Dame maintained the zebrafish used in these studies and experiments were performed with approval of the University of Notre Dame Institutional Animal Care and Use Committee (IACUC), under protocol number 19-06-5412. Materials used for these studies are as listed (Table S1). Tübingen strain WT zebrafish were used for the reported studies. Zebrafish were raised and staged as previously described (Kimmel et al., 1995). For all experiments, embryos were incubated in E3 medium at 28°C until the desired developmental stage, anesthetized with 0.02% tricaine, and then fixed using either 4% paraformaldehyde/1× PBS, or Dent's solution (80% methanol, 20% DMSO) (Gerlach and Wingert, 2014). Embryos were analyzed before sex determination, so we cannot report the effect of sex and gender in the context of this study.

### Dextran-FITC injections

*esrrγa* morphants were incubated in 0.003% phenylthiourea (Sigma-Aldrich, P7629) from 24 hpf until 96 hpf. Animals were anesthetized in 0.02% tricaine and a 40 kDa dextran-FITC conjugate (5 mg/ml) was injected intramuscularly into an embryonic somite as previously described in order to avoid disruption of the vasculature or pronephros (Kroeger et al., 2017). Embryos were imaged at 6 hpi and 48 hpi. The mean fluorescent intensity of the head and pericardium were measured using ImageJ.

### Whole-mount and fluorescent whole-mount *in situ* hybridization

WISH was performed as previously described (Marra et al., 2019a; Chambers et al., 2020a,b) with antisense RNA probes either digoxigenin-labeled (*esrrγa*, *cdh17*, *odf3b*, *slc20a1a*, *trpm7*, *slc12a1*, *slc12a3*, *jag2b*, *ptgs1*) or fluorescein-labeled (*deltaC*, *smyhc1* (referred to throughout the text as *smyhc*), *pax2a*, *odf3b*, *esrrγa*, *cdh17*, *pgc1a*, *slc12a3*) using *in vitro* transcription from IMAGE clone templates as previously described (Wingert et al., 2007; Gerlach and Wingert, 2014). Flatmounting of WISH-stained embryos was performed as previously described (Cheng et al., 2014). FISH was performed as previously described (Marra et al., 2017) using TSA Plus Fluorescein or Cyanine Kits (Akoya Biosciences). For gene expression studies, every analysis was done in triplicate for each genetic model with sample sizes of *n*>20 per replicate.

### Sectioning

Fixed zebrafish samples were exposed to 5% and 30% sucrose solution and then subjected to a 1:1 solution of 30% sucrose and tissue freezing medium (TFM). Infiltrated samples were embedded in 100% TFM and oriented in Tissue-Tek cryomolds and frozen at −80°C. Sections (14 μm) were taken on a Microm HM 550 Cryostat (Thermo Fisher Scientific) (McCampbell et al., 2014; Wesselman et al., 2023b).

### Immunofluorescence

Whole-mount IF experiments were completed as previously described (Gerlach and Wingert, 2014; Kroeger et al., 2017; Marra et al., 2017, 2019c; Chambers et al., 2020a,b). For cilia and basal bodies, anti-tubulin acetylated diluted 1:400 (Sigma-Aldrich, T6793) and anti γ-tubulin diluted 1:400 (Sigma-Aldrich, T5192) were used, respectively. Cryosectioned samples were completed as previously described (Gerlach and Wingert, 2014). For cilia and basal bodies, anti-tubulin acetylated diluted 1:1000 (Sigma-Aldrich, T6793) and anti γ-tubulin diluted 1:400 (Sigma-Aldrich, T5192). For cell polarity, animals were fixed in Dent's solution, and anti-aPKC diluted 1:500 (Santa Cruz Biotechnology, 2300359) was used to mark apical surface and anti-Na^+^K^+^ ATPase diluted 1:35 (Developmental Studies Hybridoma Bank, 528092) for a basolateral marker. See Table S1 for antibody details.

### PGE_2_ metabolite quantification

PGE_2_ metabolite quantifications were completed according to the manufacturer's protocol and adapted from previously reported methods (Cayman Chemical Company, #500141) (Esain et al., 2015; Chambers et al., 2020b). In brief, groups of 25 WT or *esrrγa* MO-injected zebrafish were pooled, anesthetized and flash frozen in 100% ethanol. Lysates were homogenized and supernatant was isolated after centrifugation at 4°C (17,000 ***g*** for 20 min). The kit reagents and manufacturer supplied protocol was followed for assay completion using a plate reader (SpectraMax ABSPlus) at 420 nm.

### Rescue experiments with dmPGE_2_

Chemical treatments were completed as previously described (Marra et al., 2019a; Poureetezadi et al., 2016; Chambers et al., 2020b). Briefly, 16,16-dmPGE_2_ (Santa Cruz Biotechnology, SC-201240) was dissolved in 100% DMSO to make 1 M stocks then diluted to the 100 µM treatment dose. Treatments were completed in triplicate with *n*>20 embryos per replicate.

### qRT-PCR

Groups of 30 zebrafish (WT, *esrrγa* morphants) were pooled at 24 hpf. Trizol (Ambion) was used to extract RNA, qScript cDNA SuperMix (QuantaBio) was used to make cDNA. PerfeCTa SYBR Green SuperMix with ROX (QuantaBio) was used to complete qRT-PCR with 100 ng for *ptgs1* and 1 ng for 18S controls being optimal cDNA concentrations. The AB StepOnePlus qRT-PCR machine was used with the following program: 2 min 50°C hold, 10 min 95°C hold, then 35 cycles of 15 s at 95°C (denaturing) and 1 min at 60°C (primer annealing) and product extension steps, respectively. Each target and source were completed in biological replicates and technical replicates, each with the median Ct value normalized to the control. Data analysis was completed by using delta delta Ct values comparing WT uninjected siblings with the respective morphant groups with 18S as a reference. Primers used to target 18S were: forward 5′-TCGGCTACCACATCCAAGGAAGGCAGC-3′ and reverse 5′-TTGCTGGAATTACCGCGGCTGCTGGCA-3′. Primers used to target *ptgs1* were: forward 5′-CATGCACAGGTCAAAATGAGTT-3′ and reverse 5′-TGTGAGGATCGATGTGTTGAAT-3′.

### cRNA synthesis, microinjections and rescue studies

The zebrafish *esrrγa* ORF was cloned into a pUC57 vector flanked by a 5′ KOZAK sequence, single BamH1, SalI and EcoRV restriction sites, and a SP6 promoter region. On the 3′ side, the ORF was followed by a series of STOP codons, a SV40 poly A tail, single NdeI, EcoRI and NotI restriction sites and a T7 promoter region. *esrrγa* RNA was generated by linearizing with Not1 and SP6 run off with the mMESSAGE mMACHINE SP6 Transcription kit (Ambion). *esrrγa* RNA was injected into WT with or without a co-injection of *esrrγa* splice-blocking morpholino at the one-cell stage at a concentration of 500 pg. The *ptgs1* ORF was cloned in to a pUC57 vector flanked by a 5′ KOZAK sequence, Cla1 restriction site and a SP6 promoter region. On the 3′ side, the ORF was followed by a series of STOP codons, an SV40 poly A tail, a NotI restriction site and a T7 promoter region. *ptgs1* RNA was generated as with *esrrγa* and injected at 900 pg.

### CRISPR-Cas9 mutagenesis

Methods were adapted from Hoshijima et al. (2019). In short, target sequences were selected using the Integrated DNA Technologies (IDT) predesign tool, and cross referenced using online program CHOP-CHOP (https://chopchop.cbu.uib.no/). Selected crRNA and tracrRNA tools were obtained (IDT), and dissolved into a 100 µM stock with duplex buffer (IDT). To form the crRNA:tracrRNA duplex, equal amounts of crRNA were combined with tracrRNA, and exposed to a rapid heat/slow cool program in a thermocycler. The 50 µM duplexed crRNA:tracrRNA was diluted to 25 µM with duplex buffer (IDT). Cas9 protein (IDT) was prepared and aliquoted according to the protocol described by Hoshijima et al. (2019). Injection mixes were prepared as follows: 1 µl 25 µM crRNA:tracrRNA (crRNA 1)+1 µl 25 µM crRNA:tracrRNA (crRNA 2)+1 µl 25 µM Cas9 protein+2 µl RNase-free water. This mixture was incubated at 37°C for 10 min and then stored at room temperature. Zebrafish embryos were injected at the one-cell stage with 2-3 nl of the 5 µM crRNA:tracrRNA:Cas9 mixture. T7 endonuclease assay was used to confirm genome editing as described (Chambers et al., 2020a). For crispant verification, primers were designed to flank both target sites (Fig. S2A). In short, DNA was prepared from individual animals and amplified via PCR. Products were gel purified with QIAquick gel extraction kit (Qiagen). Approximately 400 ng of purified product and 2 µl 10× NEB Buffer 2 (total volume 20 µl) was rehybridized in a thermocycler using the following program: 5 min at 95°C, ramp down to 85°C at 2°C/s, ramp down to 25°C at 0.1°C/s, 25°C for 10 min. Rehybridized product was digested with 1 µl T7 endonuclease I (New England Biolabs) at 37°C for 2 h and visualized on a 1.5% agarose gel.

### Genetic models

Antisense MOs were obtained from Gene Tools. MOs were solubilized in DNase/RNase free water to generate 4 mM stock solutions which were stored at 20°C. Zebrafish embryos were injected at the one-cell stage with 1-2 nl of diluted MO. Optimal dosage was determined via RT-PCR for *esrrγa* (Fig. S2B) or previously published doses (Chambers et al., 2018, 2020b). Suboptimal doses were determined by using half of the optimal dose, as prescribed by previous studies (Chambers et al., 2018, 2020b; Epting et al., 2022; Lefkopoulos et al., 2020; Lu et al., 2021). *esrrγa* was targeted with two morpholinos: a start site (ATG) morpholino: 5′-CAATGTGGCGGTCCTTGTTGGACAT-3′ (667 µM optimal), and a splice-blocking morpholino 5′-AGGGTAAAAGCCAACCTTGAATGGT-3′ (400 µM optimal, 200 µM suboptimal). The splice-blocking morpholino was validated using RT-PCR using the following primers: *esrrγa* RT-PCR forward 5′-CTGGTGCCAAGCGTTATGAGGACTGTTCCAG-3′ and *esrrγa* RT-PCR reverse 5′-GAGGCAGAGCCAGTTGAGGGTTCAAATAGG-3′. *ppargc1a* was targeted with the following validated MO: 5′-CCTGATTACACCTGTCCCACGCCAT-3′ (400 µM optimal, 200 µM suboptimal) (Bertrand et al., 2007; Chambers et al., 2018, 2020b).

### Mouse sample processing and IF

REC-ERRγ KO mice have been previously described (Zhao et al., 2018) and experiments were approved by the IACUC at the Children's Hospital of Philadelphia (CHOP). Mouse kidney paraffin sections were shipped from Dr Liming Pei's lab at CHOP to the University of Notre Dame for processing and analysis. All samples were deparaffinized with the following washes: 10 min xylene (twice), 5 min 100% ethanol (twice), 5 min 90% ethanol, 5 min 70% ethanol, 5 min 50% ethanol, 10 min water. Following deparaffinization, samples underwent heat-induced antigen retrieval in EDTA Buffer (pH 9) for 40 min at 100°C. Samples were then stored overnight in PBS. Cilia were stained using 1:400 anti-acetylated α-tubulin and secondary 1:500 anti-mouse Alexa Fluor 647 (see Immunofluorescence section). After three washes in PBS, proximal tubule was stained with 1:50 LTL-fluorescein (Vector Laboratories, FL-1321-2) in PBS for 2 h. After three washes in PBS, distal tubule was stained with 1:50 DBA-rhodamine (Vector Laboratories, RL-1332-2) in PBS for 2 h. Nuclei were stained with 1:1500 DAPI in PBS for 15 min. See Table S1 for antibody details.

### Image acquisition and phenotype quantification

A Nikon Eclipse Ni with a DS-Fi2 camera was used to image WISH samples and live zebrafish. Live zebrafish were mounted in methylcellulose with trace amounts of tricaine present. IF and FISH images were acquired using a Nikon C2 confocal microscope or a Nikon A1R confocal microscope. For mouse samples, images were obtained on the Nikon A1R confocal microscope. Images were acquired from four sections per animal. A minimum of six pictures were taken per animal, with upwards of 20 tubules included per image. For the 7-day timepoint, analysis includes 16 WT pictures and 15 KO pictures. For the 21-day timepoint, analysis includes 44 WT pictures and 47 KO pictures.

### Quantification and statistical analysis

Cilia phenotypes were quantified using ImageJ/Fiji (https://imagej.nih.gov) software tools. All measurements were completed on representative samples imaged at 60× magnification. The multi-point tool was used for counting. The segmented line tool was used for length measurements. Fluorescent intensity plots were generated with the plot profile function (Chambers et al., 2019). Each zebrafish experiment was completed in a minimum of triplicate. For mouse samples, cilia were measured using ImageJ, and only cilia that were protruding from the cell surface were included for analysis. Due to tubule dilation in CRE^+^ animals, cilia measurements were easier to record, as the cilia were more clearly visible. This resulted in more cilia measurements for CRE^+^ samples. From measurements an average and standard deviation (s.d.) were calculated, and *t*-tests or ANOVA tests were completed to compare control and experimental measurements using GraphPad Prism 9 software. All *t*-tests and ANOVA tests were unpaired/one-way, with the exception of Fig. S2F, in which paired *t*-tests were used within a treatment group and unpaired *t*-tests were used to compare wild-type groups versus morphants. GraphPad Prism 9 software. Statistical details for each experiment are located in the corresponding figure legend.

## Supplementary Material

10.1242/develop.201411_sup1 Supplementary information
