# Supplementary material for: Esrrγa regulates nephron and ciliary development by controlling prostaglandin synthesis
Source: Development. 2023 May 26;150(10):dev201411. doi: 10.1242/dev.201411 (PMC10233719; doi:10.1242/dev.201411)
Supplement: Supplementary information [file develop-150-201411-s1.pdf]

Supplement 1

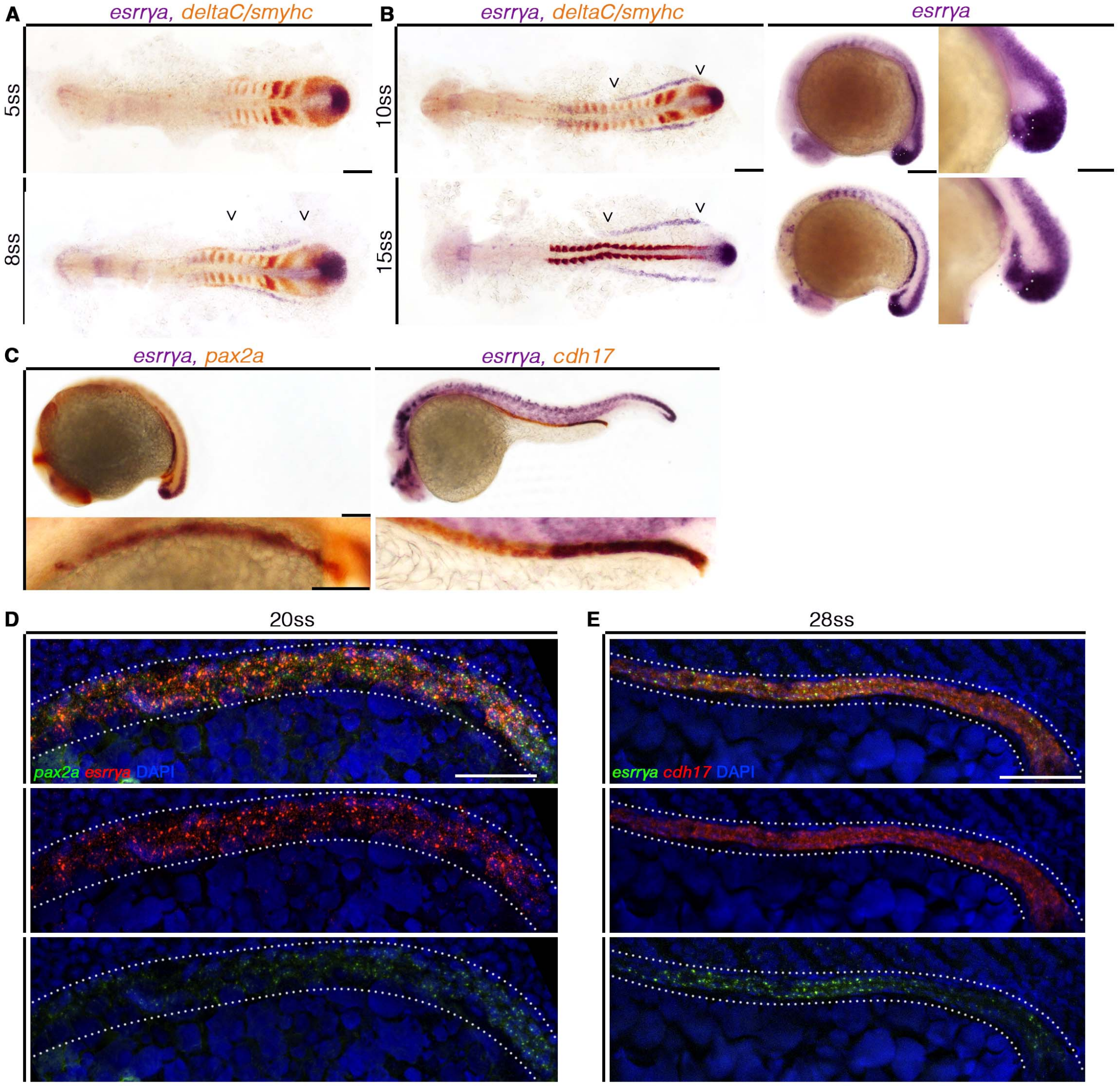

Fig. S1. (A) *esrrγa* expression (purple) and somite location (*deltaC/smyhc*, orange) stained via WISH at 5 and 8 ss. Arrow heads indicate *esrrγa* expression domain in the kidney progenitors. Scale bar = 100 µm. (B) *esrrγa* expression (purple) and somite location (*deltaC/smyhc*, orange) stained via WISH at 10 and 15 ss. Arrow heads indicate *esrrγa* expression domain in the kidney progenitors. Scale bar = 100 µm for flat mounts and scale bar = 50 µm, inset scale bar = 100 µm for whole mounts. Dotted circles surround KV expression. (C) *esrrγa* expression (purple) and essential kidney factors (*pax2a,* left and *cdh17*, right in orange) stained via WISH at 10 ss (left) and 28 ss (right). Scale bar = 50 µm, inset scale bar = 100 µm (D) FISH expression of *esrrγa* (red) and *pax2a* (green) with DAPI (blue) at the 20 ss. Top is the merged file, middle is *esrrγa* alone, and bottom is *pax2a* alone. Scale bar = 50 µm. (E) FISH expression of *esrrγa* (green) and *cdh17* (red) with DAPI (blue) at the 20 ss. Top is the merged file, middle is *cdh17* alone, and bottom is *esrrγa* alone. Scale bar = 50 µm.

Development • Supplementary information

Supplement 2

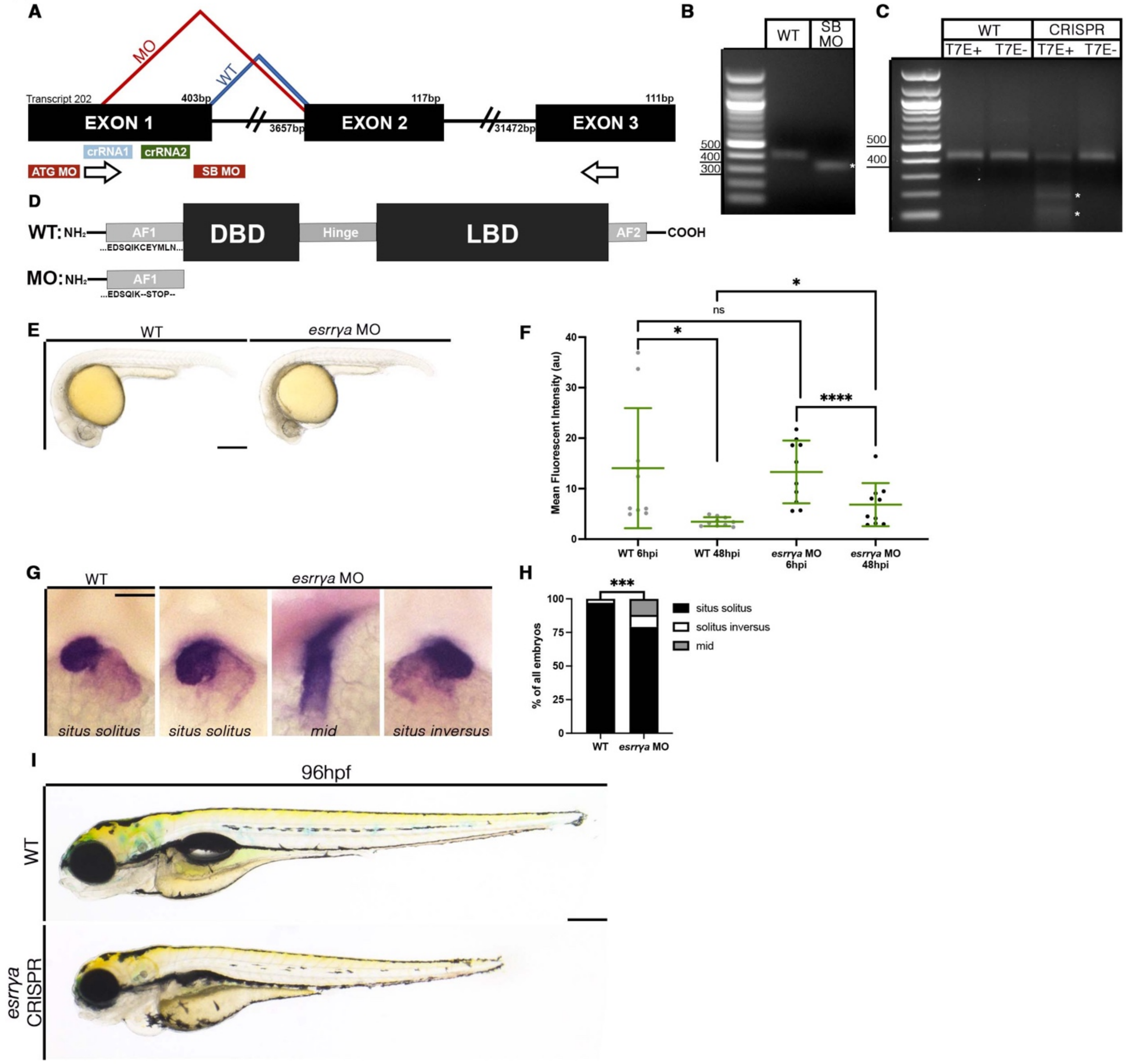

Development • Supplementary information

Fig. S2. (A) Schematic illustrating knockdown tools. Start site (ATG) MO binds to the ATG start site in exon 1. Splice blocking (SB) MO binds to the first exon-intron junction. Dark blue lines indicate WT splicing, while red lines indicate altered splicing resulting from splice blocking MO. Arrows show approximate primer locations used for RT-PCR. crRNA1 (light blue) and crRNA2 (green) indicate the relative locations of the guide RNAs used for CRISPR-Cas9 mutagenesis. (B) DNA agarose gel of RT-PCR products of WT (Lane 2) and SB MO (Lane 3) animals. WT band is present at the anticipated 450 bp length while the SB MO band (asterisk) is smaller (approximately 300 bp), indicating that part of exon 1 was spliced out. Bands were gel purified and confirmed with sequencing analysis. (C) Representative example of T7 endonuclease assay used to confirm CRISPR mutagenesis in individuals. WT animal exhibits only a single band at the expected 450 bp location, with (Lane 2) or without (Lane 3) the addition of T7 endonuclease. A CRISPR mutant exhibits the expected band at 450 bp in the absence of T7 (Lane 5), and multiple smaller bands when T7 is added (Lane 4, denoted with asterisks), suggesting mismatched base pairing and successful mutagenesis. (D) Schematic illustrating WT protein domains (top), and putative protein resulting from splice blocking MO injection (bottom). WT protein consists of activation function domains (AF1 and AF2), a DNA binding domain (DBD), a hinge domain, and a ligand biding domain (LDB). (E) Live WT (left) *esrrγa* SB MO injected (right) zebrafish embryos at 24 hpf. Scale bar = 100 µm. (F) Mean fluorescent intensity of the head region of WT and *esrrγa* SB morpholino injected animals resulting from dextran-FITC injection at 48 hpf. Measurements were taken at 6 hours post injection (hpi) and 48 hpi. Each dot represents an individual. (G) Representative images of heart looping phenotypes as determined with WISH staining of myl7. Scale Bar = 100 µm. (H) Distribution of heart looping phenotypes of WT and morphant siblings. n = 70 for each group. (I) Representative live images of WT (top) *esrrγa* cripsants (bottom). Scale bar = 200 µm. Data presented on graphs are represented as mean ± SD; * $p<0.05$, ***$p < 0.001$, and ****$p < 0.0001$ (paired t-test to compare within treatments, t-tests to compare across treatments, chi-squared analysis for heart looping data).

Development • Supplementary information

Supplement 3

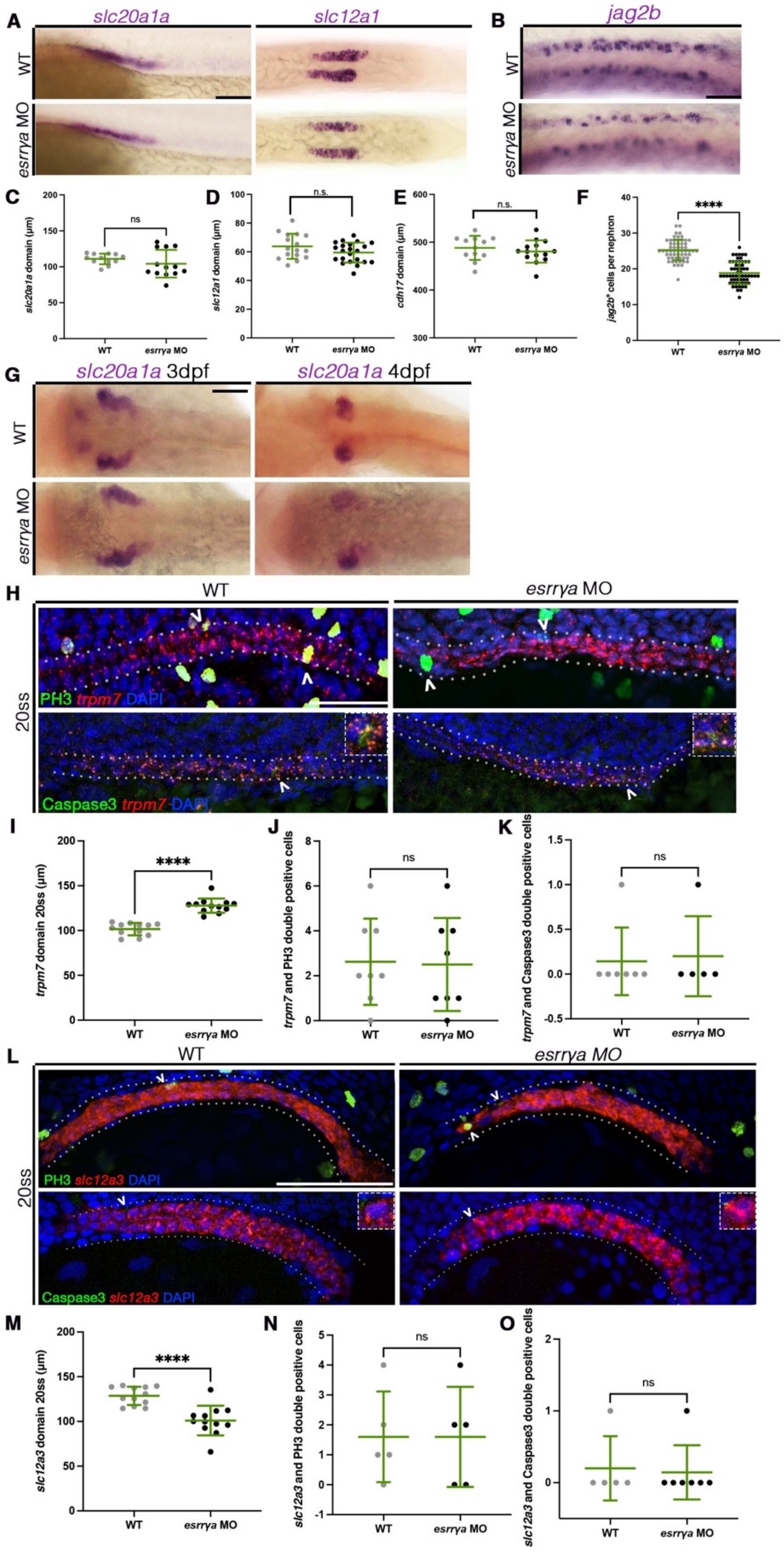

Development • Supplementary information

Fig. S3. (A) WISH of WT (top) and *esrrγa* SB MO (bottom) zebrafish at 24 hpf stained for PCT (*slc20a1a*, left), or DE (*slc12a1,* right). Scale bar = 50 µm. (B) WISH of WT (top) and *esrrγa* SB MO (bottom) zebrafish at 24 hpf stained for MCC precursors (*jag2b*). Scale bar = 50 µm. (C) PCT domain length in micrometer. Each dot represents an individual. (D) DE domain length in micrometers. Each dot represents a nephron. Two nephrons were measured per individual. (E) Entire nephron tubule length (*cdh17*) (representative image seen in Figure 1E) in micrometers. Each dot represents an individual. (F) Number of MCC precursors per nephron (*jag2b*). Each dot represents an individual. (G) Representative images of the PCT, marked by WISH of *slc20a1a*, during convolution of WT (top) and *esrrγa* SB MO (bottom) animals and 3dpf (left) and 4 dpf (right). Scale bar = 50 µm**.** (H) 20 ss WT (left) and *esrrγa* SB MO (right) nephrons (outlined with dotted line) stained for the PST (*trpm7*) via fluorescent in situ hybridization and proliferating (PH3, top) or apoptotic (Caspase3, bottom) cells via immunofluorescence. Arrow heads denote double positive cells. Scale bar = 50 µm**.** (I) PST (*trpm7*) domain length at 20 ss in micrometers. Each dot represents an individual. (J) Number of PH3 positive cells in the PST at 20 ss. Each dot represents an individual. (K) Number of Caspase3 positive cells in the PST at 20 ss. Each dot represents an individual. (L) 20 ss WT (left) and *esrrγa* SB MO (right) nephrons (outlined with dotted line) stained for the DL (*slc12a3*) via FISH and proliferating (PH3, top) or apoptotic (Caspase3, bottom) cells via immunofluorescence. Arrow heads denote double positive cells. Scale bar = 50 µm. (M) DL (*slc12a3*) domain length at 20 ss in micrometers. Each dot represents an individual. (N) Number of PH3 positive cells in the DL at 20 ss. Each dot represents an individual. (O) Number of Caspase3 positive cells in the DL at 20 ss. Each dot represents an individual. Data presented on graphs are represented as mean ± SD; ****p < 0.0001 (t-tests).

Development • Supplementary information

Supplement 4

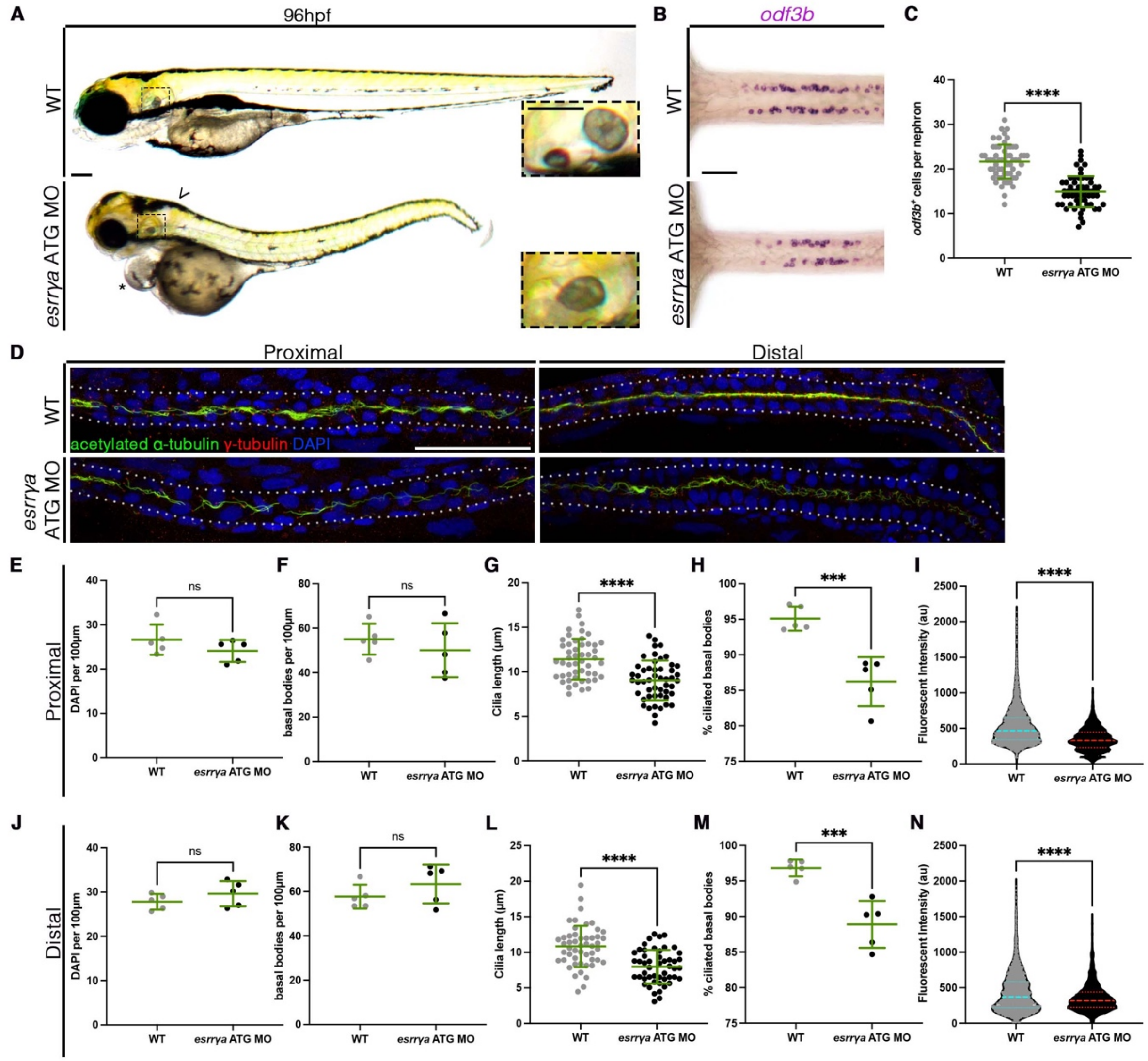

Fig. S4. (A) Live imaging of WT(top) and *esrrγa* ATG MO injected (bottom) zebrafish at 96 hpf. Morphants exhibit pericardial edema (asterisk) and fluid retention in the head (arrowhead). Inset shows fused otolith in the ATG morphants. Scale bar = 100 µm, insets = 50 µm. (B) WT (top) and *esrrγa* ATG MO injected (bottom) zebrafish embryo at 24 hpf stained with WISH for MCCs (*odf3b*). Scale bar = 50 µm. (C) Absolute number of MCCs per nephron at 24 hpf. Each dot represents a nephron. Two nephrons were measured per animal. (D) 28 hpf WT (top) and *esrrγa* ATG MO (bottom) zebrafish stained via whole mount immunofluorescence for acetylated α-tubulin (cilia, green), γ-tubulin (basal bodies, red), and DAPI (blue) in the proximal (left) and distal (right) pronephros. Scale bar = 50 µm. (E) Number of DAPI per 100 µm in the proximal pronephros. Each dot represents an individual. (F) Number of basal bodies per 100 µm in the proximal pronephros. Each dot represents an individual. (G) Cilia length in micrometers in the proximal pronephros. Each dot represents a single cilium. 10 cilia were measured per animal. WT n = 5 and *esrrγa* ATG MO n =

Development • Supplementary information

5. (H) Percentage of ciliated basal bodies (ciliated basal bodies/total basal bodies) in the proximal pronephros. Each dot represents an individual. (I) Fluorescent intensity plots (cilia, α-tubulin) for the same relative distance in the proximal pronephros at 28 hpf. Each dot represents the fluorescent intensity of an animal at a given point across the segment of interest. WT n = 5, *esrrγa* ATG MO n = 5. (J) Number of DAPI per 100 µm in the distal pronephros. Each dot represents an individual. (K) Number of basal bodies per 100 µm in the distal pronephros. Each dot represents an individual. (L) Cilia length in micrometers for the distal pronephros. Each dot represents a single cilium. 10 cilia were measured per animal. WT n = 5 and *esrrγa* ATG MO n = 5. (M) Percentage of ciliated basal bodies (ciliated basal bodies/total basal bodies) in the distal pronephros. Each dot represents an individual. (N) Fluorescent intensity plots (cilia, α-tubulin) for the same relative distance in the distal pronephros at 28 hpf. Each dot represents the fluorescent intensity of an animal at a given point across the segment of interest. WT n = 5, *esrrγa* ATG MO n = 5. Data presented on graphs are represented as mean ± SD; ***$p < 0.001$ and ****$p < 0.0001$ (t-test).

Development • Supplementary information

Supplement 5

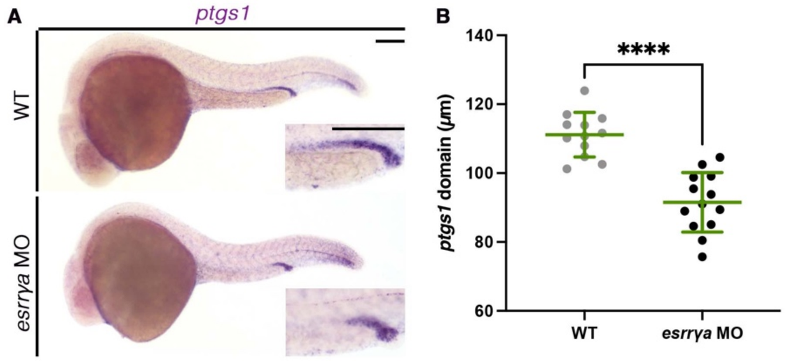

Fig. S5. (A) Expression of *ptgs1* in WT (top) and *esrrγa* SB MO (bottom) zebrafish stained via WISH at 24 hpf. Scale bar = 100 µm. (B) Length of the *ptgs1* domain in the pronephros at 24 hpf. Each dot represents an individual. Data presented on graphs are represented as mean ± SD ****p < 0.0001 (t-test).

Development • Supplementary information

Supplement 6

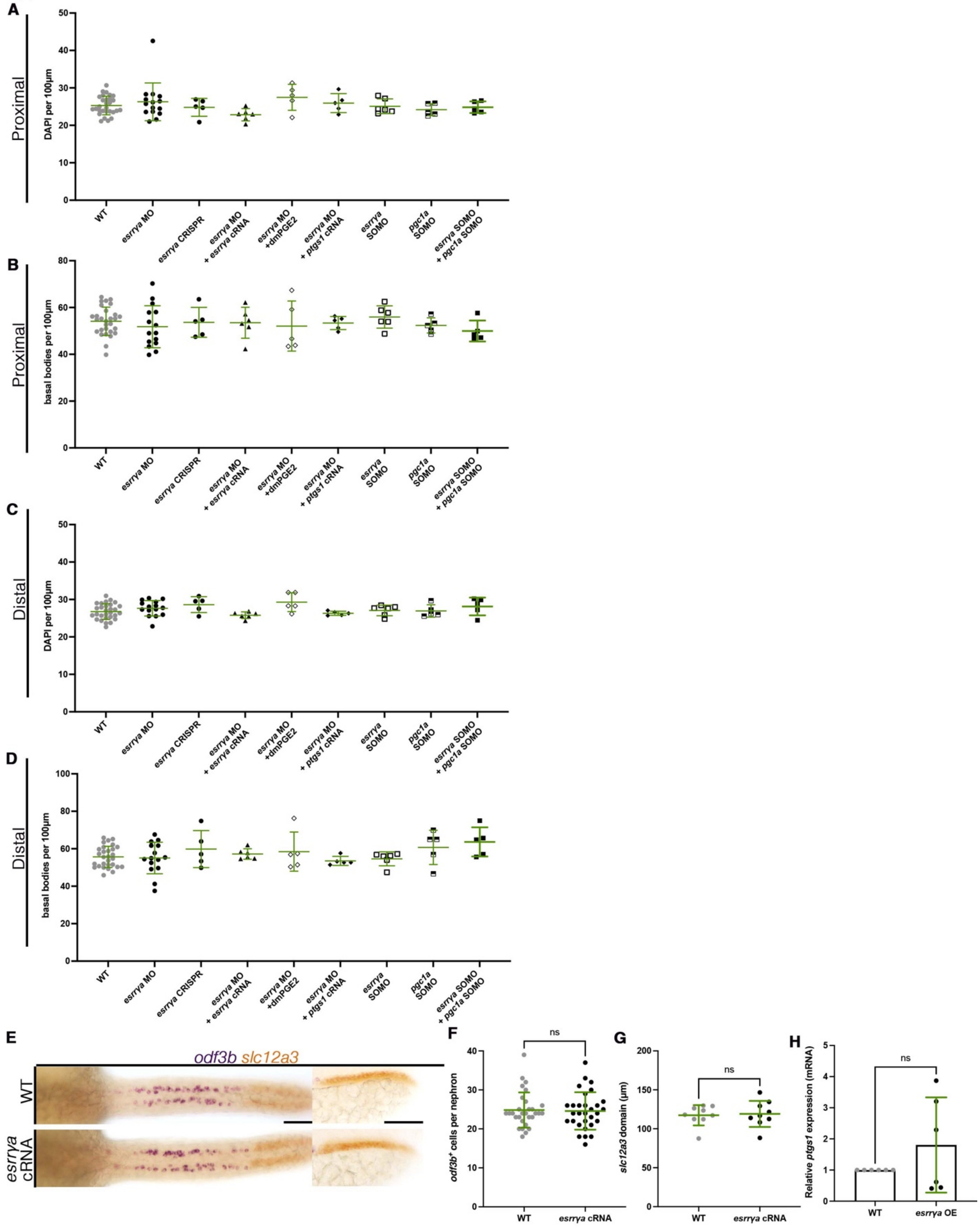

Development • Supplementary information

Fig. S6. (A) Number of DAPI per 100 μm in the proximal pronephros. Each dot represents an individual. ANOVA statistical test was not significant. (B) Number of basal bodies per 100 μm in the proximal pronephros. Each dot represents an individual. ANOVA statistical test was not significant. (C) Number of DAPI per 100 μm in the distal pronephros. Each dot represents an individual. ANOVA statistical test was not significant. (D) Number of basal bodies per 100 μm in the distal pronephros. Each dot represents an individual. ANOVA statistical test was not significant. Splice blocking morpholino was used for *esrrγa* in all panels.

Development • Supplementary information

Supplement 7

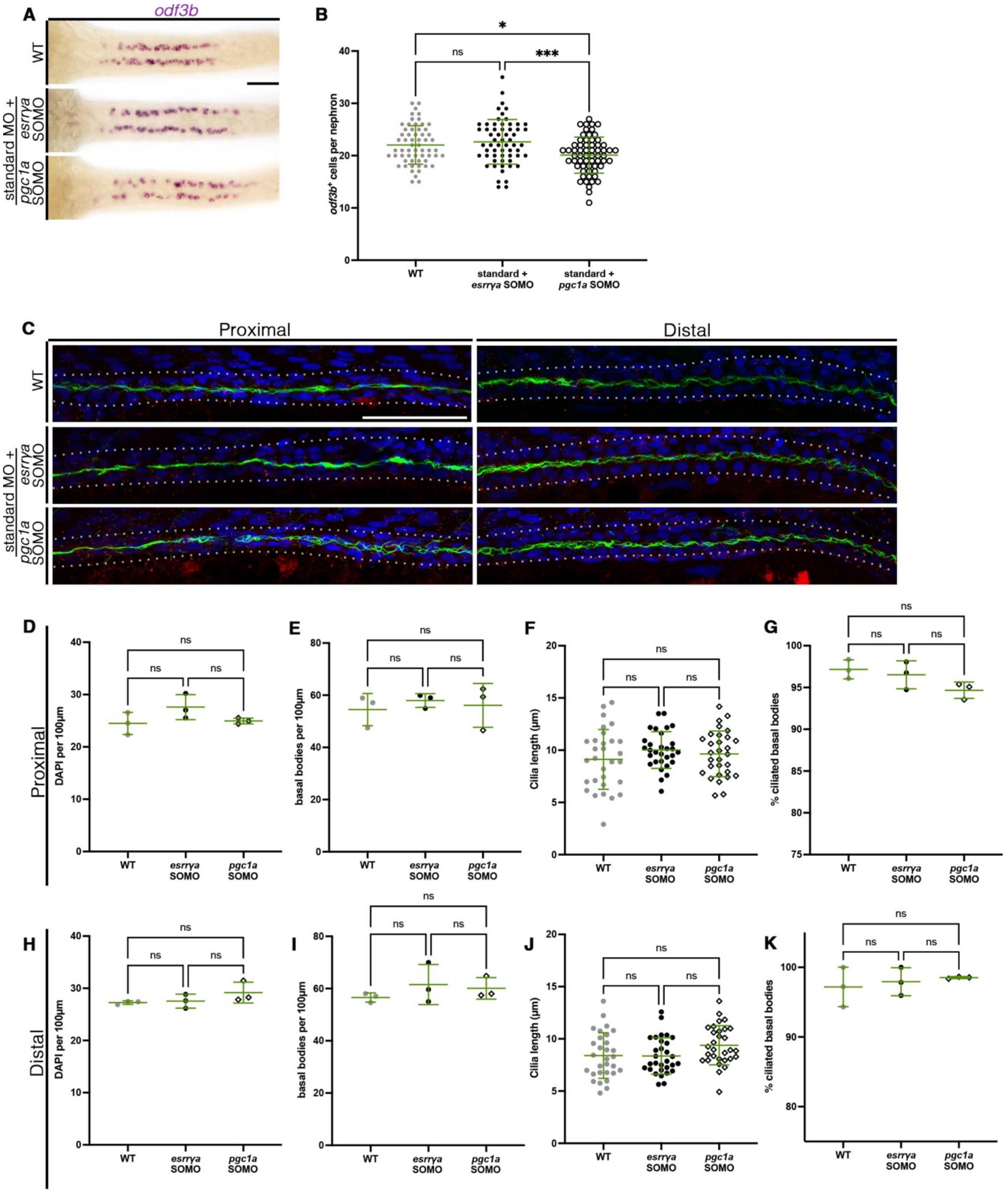

Development • Supplementary information

Fig. S7. (A) MCCs stained via WISH (*odf3b*) of 24 hpf WT, *esrrγa* sub-optimal SB MO (SOMO) co-injected with standard control MO, and *pgc1a* SOMO co-injected with standard control MO (top to bottom, respectively). Scale bar = 50 µm. (B) Number of MCCs per nephron. Each dot represents a nephron. Two nephrons were counted per individual. (D) 28 hpf WT, *esrrγa* SOMO co-injected with standard control MO, and *pgc1a* SOMO co-injected with standard control MO (top to bottom, respectively) zebrafish stained via whole mount immunofluorescence for acetylated α-tubulin (cilia, green), γ-tubulin (basal bodies, red), and DAPI in the proximal (left) and distal (right) pronephros. Scale bar = 50 µm. ((D) Number of DAPI per 100 µm in the proximal pronephros. Each dot represents an individual. (E) Number of basal bodies per 100 µm in the proximal pronephros. Each dot represents an individual. (F) Cilia length in micrometers in the proximal pronephros. Each dot represents a cilium. 10 cilia were counted per individual. For all groups n = 3. (G) Percentage of ciliated basal bodies (ciliated basal bodies/total basal bodies) in the proximal pronephros. Each dot represents an individual. (H) Number of DAPI per 100 µm in the distal pronephros. Each dot represents an individual. (I) Number of basal bodies per 100 µm in the distal pronephros. Each dot represents an individual. (J) Cilia length in micrometers for the distal pronephros. Each dot represents a cilium. 10 cilia were counted per individual. For all groups, n = 3. (K) Percentage of ciliated basal bodies (ciliated basal bodies/total basal bodies) in the distal pronephros. Each dot represents an individual. Data presented on graphs are represented as mean ± SD; * $p<0.05$ and ***$p < 0.001$ (ANOVA).

Development • Supplementary information

Supplement 8

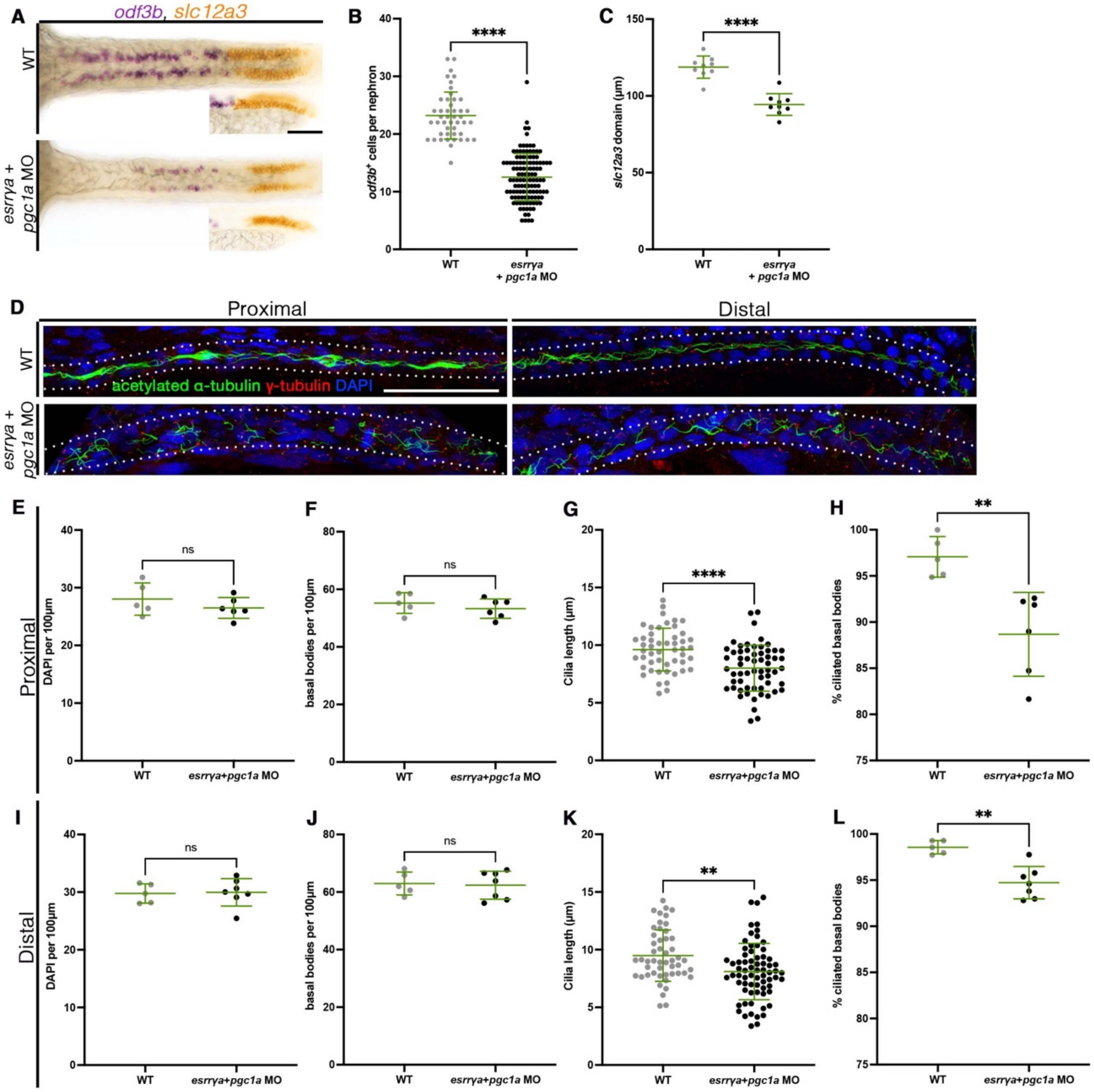

Fig. S8. (A) MCCs (*odf3b*) and DL (*slc12a3*) stained via WISH of 24 hpf WT and combination morpholino knockdown of *esrrγa* and *pgc1a*. Scale bar = 50 µm. (B) Number of MCCs per nephron. Each dot represents a nephron. Two nephrons were counted per individual. (C) Domain length of *slc12a3* in micrometers. Each dot represents an individual. (D) 28 hpf WT (top) and combination morpholino knockdown of *esrrγa* and *pgc1a* (bottom) zebrafish stained via whole mount IF for acetylated α-tubulin (cilia, green), γ-tubulin (basal bodies, red), and DAPI (blue) in the proximal (left) and distal (right) pronephros. Scale bar = 50µm. (E) DAPI per 100 µm in the proximal pronephros. Each dot represents an individual. (F) Basal bodies per 100 µm in the proximal pronephros. Each dot represents an individual. (G) Cilia length in micrometers for the proximal pronephros. Each dot represents a cilium. 10 cilia were counted per individual. WT n = 5, combination MO n = 6. (H) Percentage of ciliated basal bodies (ciliated basal bodies/total basal bodies) in the proximal pronephros. Each dot represents an individual. (I) DAPI per 100 µm in the distal pronephros. Each dot represents an individual. (J) Basal bodies per 100 µm in the distal pronephros. Each dot represents an individual. (K) Cilia length in micrometers for the distal pronephros. Each dot represents a cilium. 10 cilia were counted per individual. WT n = 5, combination MO n = 7. (L) Percentage of ciliated basal bodies (ciliated basal bodies/total basal bodies) in the distal pronephros. Each dot represents an individual. Splice blocking morpholino was used for *esrrγa* in all panels. Data presented on graphs are represented as mean ± SD; ** $p < 0.01$ and ****$p < 0.0001$ (t-test).

Development • Supplementary information

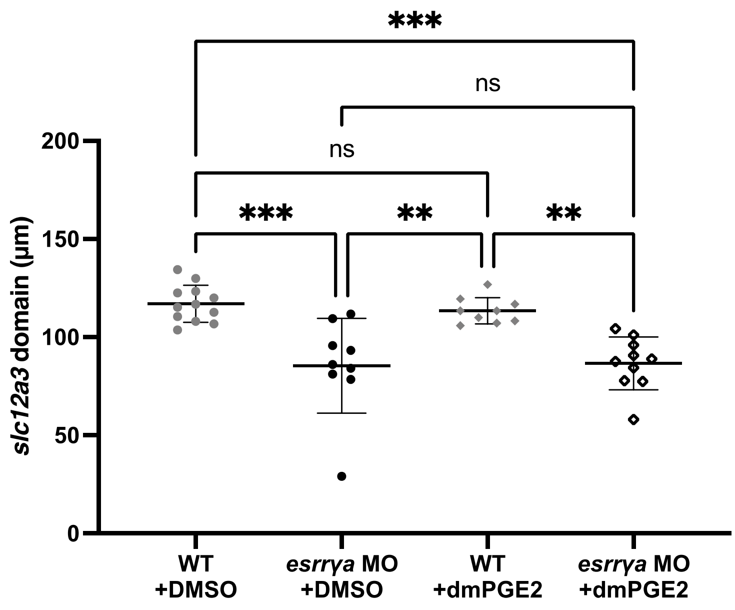

Fig. S9. DL segment length in micrometers. Each dot represents a single embryo. Data presented on graphs are represented as mean ± SD; ** p< 0.01 and ****p < 0.0001 (unpaired t-test).

Development • Supplementary information

Table S1. Experimental reagents and resources

Reagent or Resource	Source	ID
**Antibodies**		
Anti-tubulin acetylated	Sigma	T6793; RRID:AB_477585
Anti-γ-tubulin	Sigma	T5192; RRID:AB_261690
Anti-PKC	Santa Cruz	SC216; RRID:AB_2300359
Anti-Na^+K^+ ATPase	DSHB	AB_528092 RRID:AB_528092
Anti-Ph3	Millipore	06–570
Anti-Caspase3	BD Biosciences	559565
Goat anti-Mouse IgG (H+L) Highly Cross-Adsorbed Secondary Antibody, Alexa Fluor 647	Invitrogen	A21236; RRID:AB_2535805
Goat anti-Rabbit IgG (H+L) Highly Cross-Adsorbed Secondary Antibody, Alexa Fluor 647	Invitrogen	A21245; RRID:AB_141775
Goat anti-Mouse IgG (H+L) Highly Cross-Adsorbed Secondary Antibody, Alexa Fluor 568	Invitrogen	A11031; RRID:AB_144696
Goat anti-Rabbit IgG (H+L) Highly Cross-Adsorbed Secondary Antibody, Alexa Fluor 594	Invitrogen	A11037; RRID:AB_2534095
Goat anti-Mouse IgG (H+L) Highly Cross-Adsorbed Secondary Antibody, Alexa Fluor 488	Invitrogen	A11029; RRID:AB_138404
Goat anti-Rabbit IgG (H+L) Highly Cross-Adsorbed Secondary Antibody, Alexa Fluor 488	Invitrogen	A11034; RRID:AB_2576217
**Chemicals**		
16,16-Dimethyl-prostaglandin E2	Santa Cruz Biotechnology, Inc	sc-201240
40 kDa dextran-FITC	Invitrogen	D-1845
**Commercial Assays**		
Prostaglandin E2 Express ELISA Kit	Cayman Chemical	500141
mMESSAGE mMACHINE SP6 Transcription kit	Ambion	AM1340
PerfeCTa SYBR Green SuperMix with ROX	Quantabio	VWR 101414-160
qScript cDNA SuperMix	Quantabio	VWR 101414-106
TSA Plus Cyanine	Akoya Biosciences	NEL744001KT
TSA Plus Fluorescein	Akoya Biosciences	NEL741001KT
**Oligonucleotides**		
esrrγa RT-PCR forward CTGGTGCCAAGCGTTATGAGGACTGTTCCAG	This paper	N/A
esrrγa RT-PCR reverse TTGCATGTAATTTCTGGCAGTT	This paper	N/A
esrrγa CRISPR genotyping forward	This paper	N/A

Development • Supplementary information

CTGGTGCCAAGCGTTATGAGGACTGTTCCAG		
esrrγa CRISPR genotyping reverse GAGTTCAGCATGTACTCGCATT	This paper	N/A
18S qRT-PCR forward TCGGCTACCACATCCAAGGAAGGCAGC	Chambers et al., 2020	N/A
18S qRT-PCR reverse TTGCTGGAATTACCGCGGCTGCTGGCA	Chambers et al., 2020	N/A
ptgs1 qRT-PCR forward CATGCACAGGTCAAAATGAGTT	Chambers et al., 2020	N/A
ptgs1 qRT-PCR reverse TGTGAGGATCGATGTGTTGAAT	Chambers et al., 2020	N/A
esrrγa start site (ATG) morpholino CAATGTGGCGGTCCTTGTTGGACAT	Tohmé et al., 2014 Gene-Tools	ZFIN: MO1-esrrga
esrrγa splice blocking morpholino AGGGTAAAAGCCAACCTTGAATGGT	Tohmé et al., 2014 Gene-Tools	ZFIN: MO2-esrrga
ppargc1a morpholino CCTGATTACACCTGTCCCACGCCAT	Gene-Tools	ZFIN: MO1-ppargc1a
esrrγa crRNA1 TACGCTGTCCGTTAGTGAGG	IDT	predesigned guide "AA"
esrrγa crRNA2 CCCACTCTTTATGGCCCAAC	IDT	predesigned guide "AC"
Alt-R® CRISPR-Cas9 tracrRNA	IDT	1072532
**CRISPR Reagents**		
Alt-R® S.p. Cas9 Nuclease V3	IDT	1081058
Nuclease Free Duplex Buffer	IDT	11-01-03-01
**Software**		
Prism v 9	GraphPad	https://www.graphpad.com/ scientific-software/prism/
ImageJ	Fiji	ImageJ (https://imagej.nih.gov/ij/)

Development • Supplementary information
